# Pharmacological Potential and Chemical Characterization of *Bridelia ferruginea* Benth.—A Native Tropical African Medicinal Plant

**DOI:** 10.3390/antibiotics10020223

**Published:** 2021-02-23

**Authors:** Mohamad Fawzi Mahomoodally, Sharmeen Jugreet, Kouadio Ibrahime Sinan, Gokhan Zengin, Gunes Ak, Ramazan Ceylan, József Jekő, Zoltán Cziáky, Paola Angelini, Giancarlo Angeles Flores, Roberto Venanzoni, Simonetta Cristina Di Simone, Luigi Menghini, Giustino Orlando, Claudio Ferrante, Ouattara Katinan Etienne, Massimo Tacchini

**Affiliations:** 1 Department for Management of Science and Technology Development, Ton Duc Thang University, Ho Chi Minh City 758307, Vietnam; 2 Faculty of Applied Sciences, Ton Duc Thang University, Ho Chi Minh City 758307, Vietnam; 3 Department of Health Sciences, Faculty of Medicine and Health Sciences, University of Mauritius, Réduit 80837, Mauritius; bibi.jugreet2@umail.uom.ac.mu; 4 Physiology and Biochemistry Research Laboratory, Department of Biology, Science Faculty, Selcuk University, Konya 42130, Turkey; sinankouadio@gmail.com (K.I.S.); gokhanzengin@selcuk.edu.tr (G.Z.); akguneselcuk@gmail.com (G.A.); biyoram7@gmail.com (R.C.); 5 Agricultural and Molecular Research and Service Institute, University of Nyíregyháza, 4400 Nyíregyháza, Hungary; jjozsi@gmail.com (J.J.); cziaky.zoltan@nye.hu (Z.C.); 6 Department of Chemistry, Biology and Biotechnology, University of Perugia, 06100 Perugia, Italy; giancarlo.angelesflores@studenti.unipg.it (G.A.F.); roberto.venanzoni@unipg.it (R.V.); 7 Department of Pharmacy, Università degli Studi “Gabriele d’Annunzio”, via dei Vestini 31, 66100 Chieti, Italy; disimonesimonetta@gmail.com (S.C.D.S.); luigi.menghini@unich.it (L.M.); giustino.orlando@unich.it (G.O.); claudio.ferrante@unich.it (C.F.); 8 Laboratoire de Botanique, UFR Biosciences, Université Félix Houphouët-Boigny, Abidjan 00225, Côte d’Ivoire; katinan.etienne@gmail.com; 9 Department of Life Sciences and Biotechnology (SVeB), UR7 Terra&Acqua Tech, University of Ferrara, 44121 Ferrara, Italy; massimo.tacchini@unife.it

**Keywords:** *Bridelia ferruginea*, antidiabetic, anticholinesterase, antityrosinase, antioxidant, antimicrobial, antiproliferative

## Abstract

To avail the possible pharmacological actions of *Bridelia* *ferruginea* Benth., the present investigation was designed to quantitatively analyze the total flavonoid and phenolic contents and assess the various antioxidant and enzyme inhibition properties of leaf and stem bark extracts (ethyl acetate, water and methanolic) of *B. ferruginea.* Anti-proliferative effect was also investigated against human colon cancer cells (HCT116) as well as the antimicrobial potential against multiple bacterial and fungal (yeasts and dermatophytes) strains. The methanolic and water extracts of the stem bark demonstrated the highest phenolic content (193.58 ± 0.98 and 187.84 ± 1.88 mg/g, respectively), while the leaf extracts showed comparatively higher flavonoid contents (24.37–42.31 mg/g). Overall, the methanolic extracts were found to possess the most significant antioxidant potency. Compared to the other extracts, methanolic extracts of the *B. ferruginea* were revealed to be most potent inhibitors of acetyl- and butyryl-cholinesterases, tyrosinase *α*-amylase, except *α*-glucosidase. Only the ethyl acetate extracts were found to inhibit glucosidase. Additionally, the stem bark methanolic extract also showed potent inhibitory activity against *E. coli* and gram-positive bacteria (MIC (minimum inhibitory concentration): 2.48–62.99 µg/mL), as well as all the tested fungi (MIC: 4.96–62.99 µg/mL). In conclusion, *B. ferruginea* can be regarded as a promising source of bioactive compounds displaying multifunctional pharmacological activities and thus is a potential candidate for further investigations in the endeavor to develop botanical formulations for pharmaceutical and cosmeceutical industries.

## 1. Introduction

*Bridelia ferruginea* Benth. (Phyllanthaceae), a popular plant species found in the Savannah regions or rain forests of Africa [1], growing as a twisted shrub that occasionally reaches the size of a tree, is greatly used in ethnomedicine for treating various ailments in different parts of Africa [2]. In fact, *B. ferruginea* is the best studied species of the genus *Bridelia,* and its traditional uses as natural remedies have been extensively documented. These include its therapeutic uses against bladder troubles, dysentery, diabetes, rheumatic pains [3], or for wound healing [4]. *B. ferruginea* is also utilized in traditional African medicine for curing arthritis or as an embrocation to heal bruises, dislocation, burns, and boils. Nevertheless, tea prepared from the pulped bark is employed against fevers, stiffness, headaches, and as a local application for the treatment of oedemas [5]. Additionally, *B. ferruginea* bark extract is traditionally known to be used as a mouth wash, milk coagulant, vermifuge, and purgative [1].

Interestingly, numerous studies have reported extracts of *B. ferruginea* to demonstrate a range of in vivo and in vitro pharmacological activities including anti-plasmodial, anti-diarrheal, ulcer-protective, antimicrobial, anti-neuroinflammatory, and hypoglycaemic effects, amongst others [1,6,7,8]. Additionally, previous studies have revealed the presence of several phytochemicals such as flavonoids, alkaloids, tannins and cardiac glycosides, anthraquinone, phlobatinnins, and saponins in *B. ferruginea* leaf and bark [9], many of which have been found to exert important biological properties.

For instance, several phenolic compounds isolated from *B. ferruginea* stem bark were found to display radical scavenging and xanthine oxidase inhibition activities, therefore supporting the application of *B. ferruginea* in traditional medicine for treating rheumatic pains [10]. Moreover, cytotoxic constituents of *B. ferruginea* have been isolated and have shown inhibitory activity against various cancer cell lines [11]. Nevertheless, other phytochemicals from the methanol extract of *B. ferruginea* dried leaves, notably lutein and myricitrin, have demonstrated important antileishmanial and antibacterial activities, respectively [2]. Interestingly, toxicological studies of root bark hydroethanolic extract of *B. ferruginea* on rodents revealed acute (2000 and 5000 mg/kg BW) and sub-chronic (250, 500, and 1000 mg/kg BW) toxicity tests to induce neither death nor to display any significant signs of toxicity or histological changes in the organs of the animals [12].

Indeed, traditionally used medicinal plants of ethno-pharmacological relevance can be a substantial source of drugs and thus are worthy of investigation for potential biomedicine development [13]. Therefore, *B. ferruginea,* being a reputed medicinal plant, was selected in this study for analysis of the total flavonoid and phenolic contents of its leaves and stem bark extracts (ethyl acetate, water, and methanolic), together with the evaluation of their efficacy as potential enzyme inhibitors and antioxidants. A bio-pharmacological investigation was also conducted for unravelling potential applications of *B. ferruginea* extracts as anti-proliferative and antimicrobial agents. Specifically, the anti-proliferative properties were evaluated against human colon cancer (HCT116), whereas multiple pathogenic bacterial and fungal species were selected for assessing antimicrobial properties. Finally, a bioinformatics analysis was performed to investigate the putative mechanisms underlying the obtained bio-pharmacological effects.

## 2. Results and Discussion

In the current study, the quantitative analysis of the total flavonoid and phenolic contents of methanolic, water, and ethyl acetate extracts of leaves and stem bark of *B. ferruginea* was carried out using standard colorimetric assays. For instance, clearly the extracts of the leaves of *B. ferruginea* contained higher amounts of total flavonoids (24.37–42.31 mg/g) compared to the stem bark extracts (2.05–2.62 mg/g). However, the methanolic extract of the leaves showed the highest content of total flavonoids, followed by the leaves ethyl acetate and water extracts. Additionally, the extracts were found to possess relatively moderate to significant total phenolic content ranging from 26.07–193.58 mg/g. Notably, the methanolic and water extracts of the stem bark contained the highest phenolic content (193.58 ± 0.98 and 187.84 ± 1.88 mg/g, respectively), while the least phenolic contents were observed in the ethyl acetate extracts of both the leaves and stem bark, respectively (26.07 ± 0.48 and 26.90 ± 1.12 mg/g). Moreover, the methanolic and water extracts of the leaves were found to contain noteworthy phenolic levels (103.94 ± 2.00 and 85.05 ± 0.58, respectively) (Table 1). The total phenolic and flavonoid contents of *n*-hexane, ethyl acetate, and butanolic and residual aqueous fractions of *B. ferruginae* leaves have also been investigated, with *n*-hexane fraction yielding the highest phenolic and flavonoid contents [14].

Indeed, extraction procedures and solvents greatly influence the solubility of the plants’ endogenous compounds. Moreover, plant components can be non-polar or polar in nature. Particularly, phenolic compounds are more soluble in polar organic solvents owing to the presence of a hydroxyl group, and particularly, methanol is used commonly as a preferred solvent for extraction [15]. Interestingly, in the present study also, methanol was found to extract the highest phenolic and flavonoid contents from stem bark and leaves, respectively.

UHPLC/MS/MS technique was used for the chemical analysis of the different extracts, given that it provides high efficiency chromatographic separation, selectivity, and sensitivity, and the Orbitrap mass spectrometer allows precise mass measurement in order to characterize plant metabolites. UHPLC-MS analyses of all extracts were carried out in two chromatographic runs with recording of mass spectra in positive and negative ion mode, and protonated [M + H]^+^ or deprotonated [M − H]^−^ molecules and their fragments were recorded. Many compounds having phenolic hydroxyl group(s) found in the extracts were ionized preferentially in negative electrospray ionization mode (ESI). In some cases, data recorded in positive ion mode were used to characterize components. The fragmentation of flavonoids in MS/MS is structure-specific, and thus can be useful for detection and/or determination of flavonoid structures. For instance, the loss of *m*/*z* 162 indicates *O*-hexosides, whereas the loss of *m*/*z* 120 suggests C-glycosides.

The number of characterized compounds and chemical composition in methanolic and aqueous extracts of stem bark and leaves of *B. ferruginea* were similar (Table 2). Details about metabolomic analysis are provided as Supplementary Materials (Tables S1–S6; Figures S1–S7). Thirty-eight compounds from methanolic and thirty-nine compounds from aqueous extracts of stem bark were identified, while 48 and 38 compounds were characterized from methanolic and aqueous extracts of the leaves. Only 25 and 26 components were characterized from ethyl acetate extracts of stem bark and leaves (in Supplemental Materials).

Organic acids (gallic, pantothenic, kynurenic, and chlorogenic), catechins (gallocatechin, catechin, epigallocatechin, epigallocatechin-3-O-gallate, and epicatechin), flavonoids (myricetin, apigenin, quercetin, luteolin, and kaempferol) and flavone derivatives (hydroxy, methoxy, rhamnosyl, C- and O-glucosides, and rutinoside) and their isomers were assigned in the extracts.

Ellagic acid (RT: 23.94 min) and its derivatives (O-hexoside 20.34 min, O-pentoside 23.27 min, ellagic acid-4-O-rhamnoside 23.58 min, 3-O-methyl-4′-O-xyloside 25.26 min and eschweilenol A or isomer 26.39 min) could be separated and characterized. All compounds showed characteristic product ions at 300,999, corresponding to the elimination of a molecule of hexose, and O-methyl derivatives produced fragment ions, corresponding to the loss of methyl group.

A typical extracted ion chromatogram of these compounds: a: ellagic acid; b: ellagic acid-O-hexoside; c: ellagic acid-O-pentoside; d: eschweilenol C (ellagic acid-4-O-rhamnoside); ducheside A (3-O-methylellagic acid-4′-O-xyloside); and e: eschweilenol A or isomer and their MS2 spectra in negative ion mode are shown in Supplemental figures.

Phenolics and flavonoids are important antioxidant compounds present in plants. Particularly, the antioxidant activity of phenolics is attributed mainly because of their redox properties, enabling them to perform as reducing agents, in addition to donors of hydrogen atom. Furthermore, natural antioxidants are able to act as chain breakers, free-radical scavengers, complexers of pro-oxidant metal ions, as well as quenchers of singlet-oxygen formation [16]. Similarly, the potential health benefits of dietary flavonoids have been garnering appreciable interests owing to their multiple beneficial effects. Indeed, several epidemiological evidences have established the recurrent intake of flavonoid-rich foods and beverages to play a significant role in the reduction or prevention of several ailments. For instance, they are known to exhibit antiviral, antimicrobial, antiatherosclerosis, cardioprotective, anti-neoplastic, anti-ulcerogenic, antioxidant, antidiabetic, mutagenic, antiinflammatory, anti-aging, anti-hepatotoxic, hypolipidaemic, anti-hypertensive, and anti-platelet effects amongst others [17]. The mechanisms by which flavonoids display their therapeutic effects include the capacity to scavenge an array of reactive oxygen, chlorine, and nitrogen species such as superoxide as well as hydroxyl and peroxyl radicals, including hypochlorous and peroxynitrous acids. They also chelate ions, often reducing the metal ion pro-oxidant activity [18,19,20,21]. In fact, the pharmacological properties of flavonoids have been attributed to its antioxidant property [22].

Additionally, several studies have revealed the presence of a correlation between total phenolic and flavonoid contents and antioxidant capacity [23,24,25]. Importantly, it has also been reported that as antioxidants, phenolics and flavonoids are capable of exerting diverse biological effects such as antiallergic, anti-inflammatory, antidiabetic, antimicrobial, antipathogenic, antiviral, antithrombotic, and vasodilatory effects and averting disease conditions like cancer, Alzheimer’s disease, heart problems, cataracts, and eye disorders [26].

The production of highly reactive oxygen species (ROS) induces oxidative stress, which plays a fundamental role in the pathogenesis of many physiological disorders, including cell injury, cancer, and hepatic, heart, neurodegenerative, and renal disorders [15]. Thus, given the increasing risk factors of humans to different types of deadly diseases, there has been a global emerging trend towards the utility of medicinal and dietary plants as therapeutic antioxidants. In fact, an inverse relation between the dietary intake of antioxidant-rich medicinal plants and occurrence of human diseases has been established [27].

In the current work, the in vitro antioxidant potentials of the extracts were evaluated by using a total of six assays, namely DPPH, ABTS, FRAP, CUPRAC, metal chelating, and phosphomolybdenum assays (Table 3). Interestingly, the methanolic and water extracts of the leaves and stem bark displayed significant radical scavenging potential (DPPH: 95.26–491.59 mg/g; ABTS: 118.34–804.22 mg/g), in comparison to the ethyl acetate extracts of stem bark and leaves, which showed relatively low radical scavenging activity (DPPH: 31.68 ± 0.46; 10.71 ± 0.78 mg/g and ABTS: 22.98 ± 0.93; 2.97 ± 0.66 mg/g, respectively).

Remarkably, the same trend could be observed in CUPRAC and FRAP assays, whereby methanolic extracts of stem bark and leaves were significantly potent as reducing agents (CUPRAC: 1066.93 ± 12.02 and 395.81 ± 12.02 mg/g, respectively; FRAP: 633.44 ± 10.13 and 256.72 ± 3.39 mg/g, respectively), and then subsequently the water extracts of leaves and stem bark (136.26–328.08 mg/g), while the ethyl acetate extracts demonstrated comparatively lower reducing power (30.97–81.54 mg/g). Likewise, the methanolic and water extracts of stem bark demonstrated the highest total antioxidant capacity in the phosphomolybdenum assay (7.11 ± 0.39 and 6.92 ± 0.20 mmol/g, respectively). However, only moderate total antioxidant capacity was noted by the other extracts (2.03–3.17 mmol/g). Unlike in the other antioxidant assays conducted herein, the ethyl acetate extracts were exceptionally found to display the highest activity in the metal chelating assay (stem bark: 26.59 ± 0.25 and leaves: 19.57 ± 0.50 mg/g) relative to the other extracts (8.70–17.83 mg/g) (Table 3).

Other studies such as the one conducted by Olaide et al. [28] also assessed the in vitro antioxidant potentials of different extracts of stem bark of *B. ferruginea*. In contrast to the present findings, the aqueous extract in that study was found to be a better radical scavenger, showing a significantly higher percent inhibition, as opposed to that of ethanol and ethyl acetate extracts in the DPPH method. Additionally, the aqueous extract was better in Fe^2+^ chelating activity and contained higher total phenol than ethanolic and ethyl acetate extracts. The extracts were found to possess hydroxyl radical and nitric oxide scavenging activities as well [28]. Furthermore, substantial antioxidant activity was revealed for the aqueous extract of *B. ferruginea* bark, using six standard tests including ferric thiocyanate lipidic peroxidation inhibition and thiobarbituric acid reacting substances, reducing power, chelating power, and FRAP and DPPH reduction methods [29].

Interestingly, the studied extracts, particularly the methanolic and water extracts, were observed to be rich in catechin and their derivatives (Table 2). In fact, it has been reported that the number of hydroxyl groups and the presence of characteristic structural groups have a major impact on the antioxidant activity of catechins. Additionally, catechins exhibit the strong property of neutralizing reactive oxygen and nitrogen species [30]. Hence, the good antioxidant potential of extracts obtained herein could be related to the presence of these compounds. According to the cholinergic hypothesis, memory impairment induced in Alzheimer’s disease takes place because of a deficiency of cholinergic function in the brain, therefore reducing cortical and hippocampal amounts of the neurotransmitter acetylcholine (ACh) and related enzyme choline transferase. Consequently, inhibition of cholinesterases is regarded as a valuable therapeutic approach for treating symptoms in patients with Alzheimer’s disease [31]. Indeed, plants are highly rich sources of biologically active compounds, offering huge scope to modern pharmaceutical industry for drug designing. In fact, many synthetic drugs owe their origin to plant-based complementary medicine. Likewise, new treatment strategies based on medicinal plants have been established for diseases like Alzheimer’s disease, being among the most frequent cause of mortality globally [32,33].

In the present work, the methanolic extracts of the stem bark of *B. ferruginea* showed the highest anti-cholinesterase activity, followed by the methanolic leaf extracts (AChE: 5.18 ± 0.04 and 4.64 ± 0.08; BChE: 12.79 ± 0.93 and 9.27 ± 1.08, respectively) (Table 4). Nonetheless, relatively good to moderate cholinesterase inhibition was obtained by the other extracts (AChE: 2.39–4.37 mg/g; BChE: 3.15–7.70 mg/g) (Table 4). Other *Bridelia* species such as *B. speciosa* have also been reported to act as cholinesterases’ inhibitors [34].

Disorder in melanin formation has been found to cause a range of skin diseases in humans, for example hyperpigmentation, vitiligo, lentigo, and skin cancer. Additionally, the appearance of brown pigments in vegetables and fruits caused by tyrosinase activity is a leading reason for postharvest losses [35]. Tyrosinase is a multi-copper enzyme widespread in different organisms that plays an essential role in melanogenesis and enzymatic browning. Accordingly, the discovery, isolation, synthesis, and characterization of new effective tyrosinase inhibitors have been a major focus of research for different applications in food, pharmaceutical, and cosmetics industries [36].

In the present work, all the extracts were observed to inhibit tyrosinase (46.04–157.07 mg/g). In particular, the methanolic extracts could be demarcated as very potent tyrosinase inhibitors (stem bark: 157.07 ± 0.37 mg/g and leaves: 150.71 ± 0.57 mg/g), followed by water extract of stem bark and the ethyl acetate extracts (103.13–123.23 mg/g). In contrast, water extract of leaves showed the least anti-tyrosinase effect (46.04 ± 3.47 mg/g) (Table 4).

Indeed, a particularly remarkable and delicate relationship exists between melanogenesis and antioxidant defense systems, linked to scavenging of reactive oxygen species. The synergistic effect in this relationship enhances the efficiency of antioxidants in their ability to scavenge free radicals while tyrosinase inhibitors work and in consequence decreases melanin synthesis [37]. Additionally, tyrosinase inhibitory activity might rely on the hydroxyl groups of the phenolic compounds in the extracts that could form a hydrogen bond to a site of the enzyme, thereby resulting in reduced enzymatic action. On the other hand, some tyrosinase inhibitors are able to act via hydroxyl groups that bind to the active site of the enzyme, causing steric hindrance or altered conformation. However, antioxidant capacity may be one of the main reasons contributing to tyrosinase inhibition [38]. Hence, the particularly high anti-tyrosinase effect of methanolic extracts obtained in the present study could be partly due to the relatively good total phenolic content and most likely to the high antioxidant potentials demonstrated in most antioxidant assays.

The inhibition of carbohydrate-hydrolyzing enzymes such as α-amylase and α-glucosidase represents undeniably one of the important therapeutic strategies considered in the treatment of type II diabetes mellitus [39]. Hyperglycemia, which is one of the major characteristics of type II diabetes, is regarded as the principal reason for complications. A number of pharmacological interventions have been employed to enhance treatment of diabetes, using different modes of action, notably by stimulating insulin release, inhibiting gluconeogenesis, increasing the number of glucose transporters, as well as diminishing absorption of glucose from the intestine, which can be achieved with enzyme inhibitors like acarbose, miglitol, and voglibose [40]. However, gastrointestinal adverse effects render these drugs less appealing as therapeutic agents, thus making researchers consider natural therapies as viable substitutes [41], having made their mark throughout history. In fact, earlier studies have reported a number of medicinal plants and their derived compounds to possess the capability to retard enzymes involved in carbohydrate metabolism [42,43,44].

While the studied ethyl acetate extracts displayed dual inhibition on the carbohydrate hydrolyzing enzymes (amylase: 0.94 ± 0.01 and 0.91 ± 0.04 mmol/g; glucosidase: 6.68 ± 0.01 and 6.24 ± 0.29 mmol/g), the other extracts selectively inhibited amylase (0.21–1.38 mmol/g). The methanolic extracts especially, showed the highest anti-amylase effect (1.38 ± 0.03 and 1.35 ± 0.05 mmol/g), followed by the water extract of stem bark (1.06 ± 0.04 mmol/g) (Table 4).

Other reports have also studied the inhibitory effects of *B. ferruginea* extracts on these enzymes. For instance, in the study of Bakoma et al. [45], the ethyl acetate fraction of *B. ferruginea*, possessing high phenolic contents and antioxidant potential, was shown to exhibit higher inhibition activities on α-glucosidase (IC_50_ = 0.19 mg/mL) and α-amylase (IC_50_ = 0.24 mg/mL) in contrast to acetone fraction and hydroalcoholic extract having lower phenolic content with low inhibition activity (IC_50_ > 0.25 mg/mL), suggesting the phenolic compounds are likely the source of inhibition against both enzymes. Additionally, Kwon et al. [46] reported phenolic-enriched extract to display high *α*-glucosidase inhibition along with *α*-amylase inhibitory property. Additionally, Bhandari et al. [47] and Shanmugam et al. [48] evaluated the inhibitory activities of phenolic compounds against α-glucosidase and α-amylase, whereby they were found to inhibit both enzymes significantly. Moreover, in agreement with the present findings, the ethyl acetate fraction of the *B. ferruginea* stem bark in the study of Ojo et al. [49] showed *α*-glucosidase (IC_50_: 4.52 ± 0.50 mg/mL) and *α*-amylase (IC_50_: 5.42 ± 1.10 mg/mL) inhibitory activities. Additionally, enzymes kinetics studies pointed out that the ethyl acetate fraction acted as a non-competitive inhibitor and a competitive inhibitor for glucosidase and amylase, respectively. The presence of phenols such as 2,2′ oxydiphenol and p-hydroxyphenyl ether were suggested to be the main bioactive compounds accountable for the enzyme inhibition activities [49]. In furtherance, in a study by Mahomoodally et al. [34], another species of the *Bridelia* genus, *Bridelia speciosa* Müll. Arg. stem bark extracts, were investigated. Similarly, only the ethyl acetate extract showed inhibition against glucosidase, which was in accordance with the present findings.

**Figure 1:**
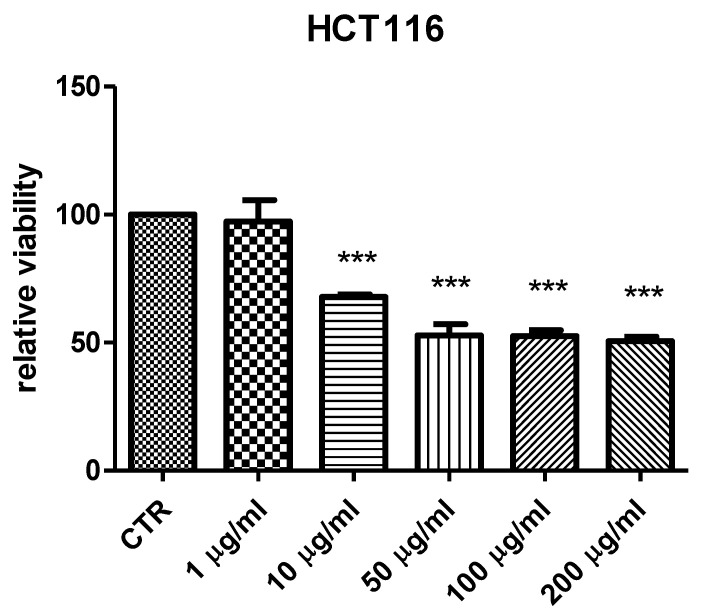
Antiproliferative effect induced by *Bridelia ferruginea* methanolic extract (1–200 µg/mL) against human colon cancer HCT116 cell line. ANOVA, *p* < 0.0001, *** *p* < 0.001 vs. Control (CTR) group.

**Figure 2:**
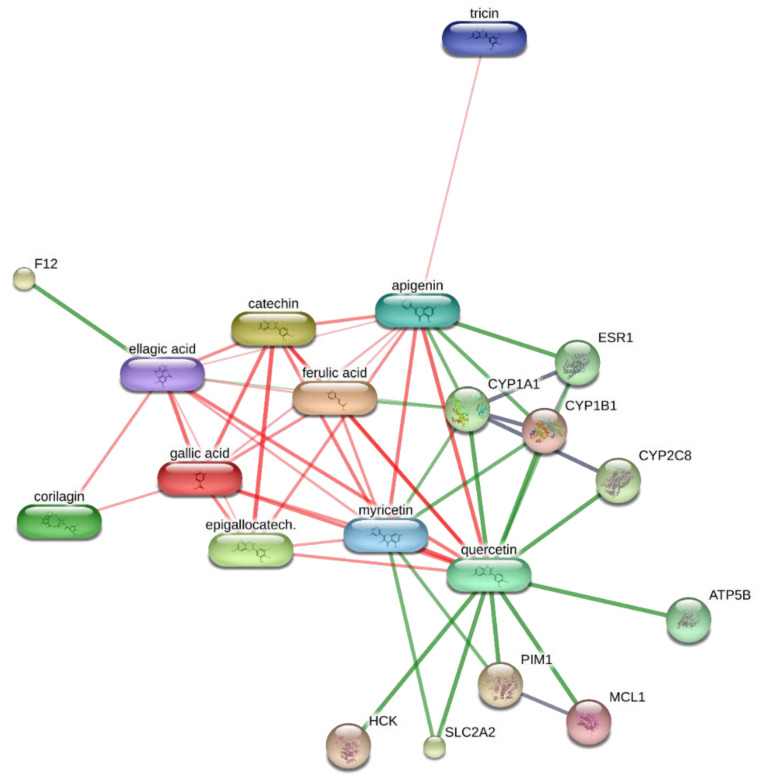
Components–targets analysis built through the bioinformatics STITCH platform that predicted interactions between extracts’ phytochemicals (quercetin, gallic acid, ellagic acid, catechin, apigenin, corilagin, epigallocatechin, myricetin, ferulic acid, tricin) and human multiple protein classes, including kinases and cytochromes.

**Figure 3:**
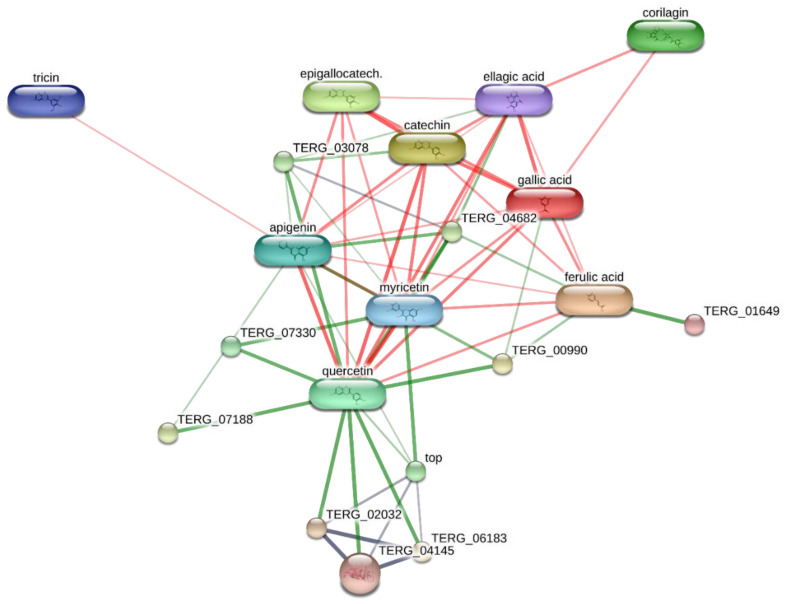
Components–targets analysis built through the bioinformatics STITCH platform that predicted putative interactions between extracts’ phytochemicals (quercetin, gallic acid, ellagic acid, catechin, apigenin, corilagin, epigallocatechin, myricetin, ferulic acid, tricin) and different dermatophytes (*T. rubrum*) enzymes.

Considering the potential cytotoxicity of natural compounds extracted from *B. ferruginea* [11] and the intrinsic antimicrobial activity displayed by herbal extracts rich in total phenolic compounds [50,51], the present study also attempted to inspect the anti-proliferative and antimicrobial effects of *B. ferruginea.* The anti-proliferative properties were evaluated against the human colon cancer HCT116 cell line. While antimicrobial effects were assayed against multiple Gram− (*E. coli*, *P. aeruginosa*, *S. tiphy*), Gram+ (*B. subtilis*, *B. cereus*, and *S. aureus*), and fungi (*C. albicans, C. tropicalis* and *C. parapsilosis*) strains, deeply involved in colon inflammation [37,52,53,54]. Antimycotic properties were also evaluated against different dermatophytes strains (*T. mentagrophytes*, *T. tonsurans, T. rubrum, A. quadrifidum*, *N. gypseum*, *A. currei*, and *A. insingulare*). Specifically, the bio-pharmacological evaluations focused on the methanolic extract from stem bark that displayed the highest scavenging/reducing and enzyme inhibition properties. Initially, the biocompatibility limit of the extract was determined, in the concentration range of 0.1–20 mg/mL, through the brine shrimp *A. salina* lethality test, an eco-toxicological assay regarded to be, at least partly, predictive of cytotoxicity [55]. The LC_50_ value resulting from brine shrimp test was <2 mg/mL. Therefore, at least ten-fold lower concentration was employed for the subsequent in vitro tests. Specifically, a concentration-dependent (1–200 µg/mL) inhibition of HCT116 cell viability was observed (Figure 1), thus demonstrating an anti-proliferative effect that occurs within the range of biocompatibility yielded by the brine shrimp test. Recently, polar extracts from *B. speciosa* were found to reduce the viability of liver cancer cells (HepG2) [34]. The inhibition of cancer cell viability was related, at least in part, to the catechin fraction measured in the extracts. In analogy, it can be hypothesized that the current inhibitory effect induced by *B. ferruginea* stem bark methanolic extract on HCT116 cell viability could depend, albeit partially, on the numerous catechin compounds detected by HPLC-MS qualitative analysis. This hypothesis is also substantiated by literature data highlighting the inhibitory effects of HCT116 cell viability induced by epigallocatechin-3-gallate, possibly mediated by the inhibition of MET receptor tyrosine kinase and DNA methyltransferases [56,57]. Catechin levels have also been related to the anti-proliferative effects induced by anti-inflammatory *Epilobium angustifolium* and *Phyllantus niruri* water extracts, in prostate cancer PC3 cells [50]. However, the bioinformatics prediction based on the chemical composition of *B. ferruginea* methanolic extract, conducted via the STITCH platform (http://stitch.embl.de/ accessed on 15 February 2021), pointed to the prominent position of quercetin in the scenario of the components–targets analysis (Figure 2). In particular, quercetin was predicted to interact with pim-1 oncogene (PIM-1), a proto-oncogene involved in cell survival and proliferation [58], whose expression was also observed in HCT116 cells [59]. Overall, the past and present studies suggest the polar extracts from *Bridelia* species as good candidates for future studies aimed to explore anticancer activity, in vivo. Regarding the antibacterial activity, the methanolic extract was effective against all the tested strains, except *P. aeruginosa.* Intriguingly, the bacteriostatic effects induced by the extract occurred at concentrations (2.48–125 µg/mL) somewhat lower than the biocompatibility limit calculated by the brine shrimp test and in the concentration range effective in inducing the anti-proliferative effect on HCT116 cells (Table 5). This suggests that the tested extract could also be a promising agent for counteracting dysbiosis-related inflammatory disorders in the colon. Among the assayed strains, *E. coli* was the most sensitive to the bacterial growth inhibition induced by the extract that was also effective against all tested fungi and yeasts strains. Among the fungi, dermatophytes species showed higher sensitivity to the growth inhibitory effects induced by the extract (Table 6). The inhibition of dermatophytes growth adds to the highest anti-tyrosinase effect (Table 4) displayed by this extract, thus further supporting potential uses against skin hyperpigmentation, which could be also stimulated by dermatophytes infections [60,61]. Currently, the observed antimicrobial effects are consistent, albeit in part, with the presence of phenols and flavonoids [62,63]. Interestingly, the antibacterial mechanism and in particular, membrane disruption, in both Gram-positive and Gram-negative bacteria, that contributes to the antibacterial activity of most plant phenolics has been widely assessed, as reviewed by Rempe et al. [64]. The present HPLC-MS fingerprint analysis and recent published data suggest that catechin fraction could play a pivotal role in the observed antibacterial effects, especially against *E. coli* [50,65]. However, the bioinformatics prediction conducted to investigate the putative interactions of extracts’ phytocompounds against the dermatophytes species *T. rubrum*, chosen for its availability on the bioinformatics platform STITCH, suggested the interactions of epigallocatechin, quercetin, and gallic acid with multiple dermatophytes enzymes, including ATP synthase (TERG_07188), UDP-glucose:sterol glucosyltransferase (TERG_00990) and xanthine dehydrogenases (TERG_06183, TERG_04145, TERG02032) (Figure 2 and Figure 3), involved in the energetic metabolism. Overall, the reported bioinformatics prediction is consistent with the antifungal effects induced by the extract. Nevertheless, future investigations are essential to quantify the identified compounds in the extract and to confirm the present findings with independent biological models.

## 3. Materials and Methods

### 3.1. Plant Material Used and Extracts’ Preparation

*Bridelia ferruginea* Benth. leaves and stem barks were collected from the village of Akpéssékro, district of Yamoussoukro (Côte d’Ivoire), in 2019, which was authenticated by the botanist Ouattara Katinan Etienne (Université Félix Houphouët Boigny, Abidjan, Côte d’Ivoire). The stem barks and leaves were cautiously separated and dried in shade for ten days, followed by grinding by a laboratory mill.

Powdered plant materials (5 g) were macerated with methanol or ethyl acetate (100 mL) for 24 h at 25 °C. After that, the solvents were evaporated by a rotary evaporator. Regarding water extracts, 5 g of the plant materials were kept in 100 mL of boiling water for 15 min, which were then filtered and lyophilized. Obtained extracts were placed for storage at 4 °C until further analysis.

### 3.2. Spectrophotometric Assays for Total Phenolics and Flavonoids

Folin–Ciocalteu method and AlCl_3_ assays were used for measurement of total phenolics contents (TPC) and flavonoid contents (TFC) [66]. Gallic acid (mg GAEs/g extract) and rutin (mg REs/g extract) were used as standards for respective assays.

### 3.3. Chromatographic Separation

Chromatographic separation was accomplished with a Dionex Ultimate 3000RS UHPLC instrument, equipped with Thermo Accucore C18 (100 mm × 2.1 mm i.d., 2.6 μm) analytical column for separation of compounds. Water (A) and methanol (B) containing 0.1% formic acid were employed as mobile phases, respectively. The total run time was 70 min; the elution profile and all exact analytical conditions have been published [67].

### 3.4. Antioxidant and Enzyme Inhibition Assays

Some antioxidant assays including metal chelating, reducing power (FRAP and CUPRAC), DPPH, ABTS, and phosphomolybdenum assays were selected for antioxidant properties. Trolox and EDTA (only for metal chelating) were used as standards. The enzyme inhibition properties were assayed by using cholinesterases, amylase, glucosidase, and tyrosinase. Both antioxidant and enzyme inhibitory assays’ experimental procedure are described in our earlier paper [68,69].

### 3.5. Artemia salina Lethality Test

The *Artemia salina* lethality test was conducted in order to predict the cytotoxicity of the *B. ferruginea* extract, in the concentration range 0.1–20 mg/mL. The details about experimental conditions are fully listed in one of our previous studies [50].

### 3.6. Cell Cultures and Viability Test

The anti-proliferative effect of *B. ferruginea* extract (1–200 µg/mL) was assayed on the human colon cancer HCT116 cell line. The viability of HCT116 was measured through the 3-(4,5-dimethylthiazol-2-yl)-2,5-diphenyltetrazolium bromide (MTT) test. The details about experimental conditions are fully listed in one of our previous studies [70].

### 3.7. Antibacterial and Antifungal Activities

The antimicrobial effects of *B. ferruginea* extract were assayed against numerous bacterial and fungal strains, according to previous studies [50,51]. Specifically, the bacterial species considered for testing the bacteriostatic effects were *E. coli* (ATCC 10536), *E. coli* (PeruMycA 2), *E. coli* (PeruMycA 3), *P. aeruginosa* (PeruMycA 5), *S. typhy* (PeruMycA 7), *B. cereus* (PeruMycA 4), *B. subtilis* (PeruMycA 6), and *S. aureus* (ATCC 6538). MIC determination was performed according to the broth dilution method M07-A9 drafted by the Clinical and Laboratory Standard Institute. Shortly, working bacterial suspensions (inocula) for MIC determination were prepared as follows: a few colonies from 24 h-old cultures on TSA plates were transferred to Mueller–Hinton broth (MHB) and incubated statically overnight at 37 °C. Cell density of each inoculum was hence adjusted to that of the opacimetric standard Mac Farland 0.5 (1.5 × 108 CFU/mL). Then, 20 μL of bacterial suspensions were used to inoculate 1 mL of MHB medium containing serial dilutions of active plant extracts. To further assess the viability of bacterial cells at MIC end-points, the tetrazolium salt assay was used. Following 20 h incubation for MIC determination, 230 μL of bacterial cultures were collected and transferred to 96-wells plates. Hence, 20 μL of a 2,3,5-triphenyl-tetrazolium chloride (TTC) solution was added to each well in order to reach a final concentration of 0.4%. Controls consisted of MHB-grown bacterial cultures (viability controls) and uninoculated MHB with plant extracts/essential oil (incubation controls). Then, 96-well plates were incubated for 6 h at 37 °C prior to measure absorbance at 405 nm in a Tecan Infinite 200 PRO spectrophotometer (Tecan Trading AG, Bern, Switzerland). As for the antifungal effects induced by the extract, the following *Candida* and dermatophytes species were considered for the assays: *C. tropicalis* (DBVPG 6184), *C. albicans* (DBVPG 6379), *C. parapsilopsis* (DBVPG 6551), *C. albicans* (DBVPG 6183), *T. mentagrophytes* (CCF 4823), *T. tonsurans* (CCF 4834), *T. rubrum* (CCF 4933), *A. quadrifidum* (CCF 5792), *N. gypseum* (CCF 6261), *A. currei* (CCF 5207), and *A. insingulare* (CCF 5417). Susceptibility testing against yeasts and filamentous fungi was performed according to the CLSI M27-A3 and M38-A2 protocols, respectively. RPMI (Roswell Park Memorial Institute) 1640 medium (Sigma) with L-glutamine and without sodium bicarbonate, supplemented with 2% glucose (*w*/*v*), buffered with 0.165 mol/L morpholinepropanesulphonic acid (MOPS), pH 7.0, was used throughout the study. Briefly, the inoculum suspensions were prepared from seven-day-old cultures grown on Sabouraud Dextrose Agar (SDA; Difco) at 25 °C and adjusted spectrophotometrically to optical densities that ranged from 0.09 to 0.11 (Mac Farland standard). Filamentous fungi and yeasts’ inoculum suspensions were diluted to a ratio of 1:50 in RPMI 1640 to obtain twice an inoculum size ranging from 0.2 to 0.4 × 10^4^ CFU/mL. This was further confirmed by plating serial dilutions of the inoculum suspensions on SDA. For the plant extracts, the MIC end-points were defined as the lowest concentration that showed total growth inhibition. The MIC end-points for fluconazole were defined as the lowest concentration that inhibited 50% of the growth when compared with the growth control. Geometric means and MIC ranges were determined from the three biological replicates to allow comparisons between the activities of plant extracts.

### 3.8. Bioinformatics

The bioinformatics analysis was conducted through the STITCH platform (http://stitch.embl.de/cgi/network.pl accessed on 15 February 2021). Specifically, a components-targets analysis was built considering the phytochemical composition of *B. ferruginea* extracts and microbial and human proteins putatively targeted by the secondary metabolites identified through UHPLC analysis.

### 3.9. Statistical Analysis

The statistical analyses related to in vitro studies were conducted through the GraphPad Prism software (5.01). The results were analyzed through analysis of variance (ANOVA), followed by the Newman–Keuls comparison multiple test. The values were considered statistically significant for *p* values less than 0.05.

## 4. Conclusions

The methanolic and water extracts of stem bark *B. ferruginea* contained the highest phenolic content (193.58 and 187.84 mg GAE/g), while the leaves extracts were observed to contain comparatively higher flavonoid content (24.37–42.31 mg RE/g) compared to the stem bark extracts (2.05–2.62 mg RE/g). From the antioxidant assays, the methanolic extract of stem bark, followed by that of the leaves, was noted to exhibit the best free radical scavenging and reducing capacity (491.59–1066.93 mg TE/g). Remarkably, the same trend was observed for most of the enzyme inhibitory potencies of the extracts. Notably, the methanolic extracts were distinguished as the strongest inhibitors of amylase, tyrosinase, and acetyl- and butyryl-cholinesterase, except glucosidase. In fact, only the ethyl acetate extracts dually inhibited both carbohydrate hydrolyzing enzymes (0.91–6.68 mmol ACAE/g). The exceptional activities of the methanolic extracts as potent antioxidants and enzyme inhibitors reported herein could principally be associated to the high phenolic or flavonoid contents detected. The anti-proliferative and antimicrobial effects induced by the methanolic stem bark extract against HCT116 cells (tested at a concentration range of 1–200 µg/mL), the bacteria (MIC of most susceptible bacteria: 2.48–62.99 µg/mL), and fungi (MIC: 4.96–62.99 µg/mL), respectively, could also be related to its rich phenolic content. Hence, the findings geared from the present study importantly highlighted the significance of this medicinal plant as a reservoir of bioactive metabolites and exhibiting manifold pharmacologic actions against key diseases such as diabetes, skin pigmentation and neurodegenerative disorders, cancer, and microbial infections. Nevertheless, while this study preliminarily amassed some important scientific data, further in vivo and clinical investigations could help to better understand the toxicity and safety profiles of this species before its applications as mainstream medicine.

## Figures and Tables

**Table 1:** Total phenolic (TPC) and flavonoid (TFC) contents of the tested extracts.

Parts	Solvents	TPC (mg GAE/g)	TFC (mg RE/g)
Leaves	EA	26.90 ± 1.12 ^e^	29.71 ± 0.82 ^b^
	MeOH	103.94 ± 2.00 ^c^	42.31 ± 0.39 ^a^
	Water	85.05 ± 0.58 ^d^	24.37 ± 0.13 ^c^
Stem bark	EA	26.07 ± 0.48 ^e^	2.62 ± 0.23 ^d^
	MeOH	193.58 ± 0.98 ^a^	2.45 ± 0.35 ^d^
	Water	187.84 ± 1.88 ^b^	2.05 ± 0.17 ^d^

Values are reported as mean ± S.D. EA: ethyl acetate; MeOH: methanolic; GAE: gallic acid equivalent; RE: rutin equivalent. Different letters (a–e) indicate significant differences in the tested extracts (*p* < 0.05).

**Table 2:** Chemical composition of *B. ferruginea* extracts.

No.	Name	Formula	Stem Bark-EA	Stem Bark-MeOH	Stem Bark-Water	Leaves-EA	Leaves-MeOH	Leaves-Water
1	Epigallocatechin-(7-O-4′)-gallocatechin or isomer	C_30_H_26_O_13_	−	+	−	−	−	−
2 ^1^	Gallic acid (3,4,5-Trihydroxybenzoic acid)	C_7_H_6_O_5_	+	+	+	−	+	+
3	Gallocatechin	C_15_H_14_O_7_	+	+	+	−	+	+
4	Pantothenic acid	C_9_H_17_NO_5_	+	+	+	−	+	+
5	Epigallocatechin-(7-O-4′)-gallocatechin or isomer	C_30_H_26_O_13_	−	−	+	−	−	−
6	Prodelphinidin C	C_30_H_26_O_13_	−	+	+	−	−	−
7	Epigallocatechin-(7-O-4′)-gallocatechin or isomer	C_30_H_26_O_13_	−	+	+	−	−	−
8	Kynurenic acid	C_10_H_7_NO_3_	−	−	−	−	+	+
9 ^1^	Catechin	C_15_H_14_O_6_	−	+	+	−	+	+
10 ^1^	Epigallocatechin	C_15_H_14_O_7_	+	+	+	−	+	+
11	Epigallocatechin-(7-O-4′)-gallocatechin or isomer	C_30_H_26_O_13_	−	+	+	−	−	−
12 ^1^	Chlorogenic acid (3-O-Caffeoylquinic acid)	C_16_H_18_O_9_	−	−	−	+	+	+
13 ^1^	Epigallocatechin-3-O-gallate (Teatannin II)	C_22_H_18_O_11_	+	+	+	−	+	−
14	5-O-(4-Coumaroyl)quinic acid	C_16_H_18_O_8_	−	−	+	−	−	−
15	Dihydrokaempferol-O-hexoside	C_21_H_22_O_11_	−	+	+	−	−	−
16 ^1^	Epicatechin	C_15_H_14_O_6_	+	+	+	−	+	+
17	Corilagin	C_27_H_22_O_18_	+	+	+	−	−	−
18	5-O-Feruloylquinic acid	C_17_H_20_O_9_	−	−	+	−	−	−
19 ^1^	Epicatechin-3-O-gallate	C_22_H_18_O_10_	−	+	+	−	−	−
20 ^1^	Ferulic acid	C_10_H_10_O_4_	+	+	+	−	−	−
21	Ellagic acid-O-hexoside	C_20_H_16_O_13_	+	+	+	−	−	−
22	Myricetin-O-hexoside	C_21_H_20_O_13_	−	−	−	+	+	+
23 ^1^	Vitexin (Apigenin-8-C-glucoside)	C_21_H_20_O_10_	−	−	+	+	+	+
24	Methylellagic acid-O-hexoside	C_21_H_18_O_13_	+	+	+	−	−	−
25	Myricitrin (Myricetin-3-O-rhamnoside)	C_21_H_20_O_12_	−	+	+	−	+	−
26	Isovitexin (Apigenin-6-C-glucoside)	C_21_H_20_O_10_	+	+	+	+	+	+
27	Coatline A or isomer	C_21_H_24_O_10_	−	+	+	−	+	−
28	Hexahydroxy(iso)flavanone	C_15_H_12_O_8_	−	−	−	−	+	+
29	Ellagic acid-O-pentoside	C_19_H_14_O_12_	+	+	+	−	−	−
30 ^1^	Rutin (Quercetin-3-O-rutinoside)	C_27_H_30_O_16_	−	−	−	+	+	+
31	Eschweilenol C (Ellagic acid-4-O-rhamnoside)	C_20_H_16_O_12_	+	+	+	−	−	−
32	Ellagic acid	C_14_H_6_O_8_	+	+	+	−	+	+
33	Methoxy-pentahydroxy(iso)flavone-O-rhamnosylhexoside isomer 1	C_28_H_32_O_17_	−	−	−	−	+	+
34	Quercetin-O-malonylhexoside	C_23_H_22_O_13_	−	−	−	−	+	+
35	Methoxy-pentahydroxy(iso)flavone-O-rhamnosylhexoside isomer 2	C_28_H_32_O_17_	−	−	−	−	+	+
36 ^1^	Myricetin (3,3′,4′,5,5′,7-Hexahydroxyflavone)	C_15_H_10_O_8_	−	+	+	−	+	+
37	Kaempferol-O-rhamnosylhexoside	C_27_H_30_O_15_	−	−	−	−	+	+
38	Tricin-O-hexoside	C_23_H_24_O_12_	−	+	−	−	−	−
39	Tricin-O-hexoside isomer 1	C_23_H_24_O_12_	−	−	−	+	+	+
40^1^	Quercitrin (Quercetin-3-O-rhamnoside)	C_21_H_20_O_11_	−	−	−	+	+	+
41	N-trans-Feruloyltyramine	C_18_H_19_NO_4_	−	−	−	−	+	−
42	Ducheside A (3-O-Methylellagic acid-4′-O-xyloside)	C_20_H_16_O_12_	+	+	+	−	−	−
43	Kaempferol-3-O-rutinoside (Nicotiflorin)	C_27_H_30_O_15_	−	−	−	+	+	+
44	Dimethoxy-tetrahydroxy(iso)flavone-O-hexoside	C_23_H_24_O_13_	+	+	+	+	+	+
45	Tricin-O-hexoside isomer 2	C_23_H_24_O_12_	−	−	−	+	+	+
46	Trihydroxy-trimethoxy(iso)flavone-O-hexoside isomer 1	C_24_H_26_O_13_	−	−	−	+	+	+
47	Isorhamnetin-3-O-rutinoside (Narcissin)	C_28_H_32_O_16_	−	−	−	+	+	+
48	3-O-Methylellagic acid-O-rhamnoside	C_21_H_18_O_12_	+	+	+	−	−	−
49	Dihydroxy(iso)flavone-C-hexoside	C_21_H_20_O_9_	−	+	+	−	+	+
50	Pentahydroxy(iso)flavanone	C_15_H_12_O_7_	−	−	−	−	+	+
51	3-O-Methylellagic acid	C_15_H_8_O_8_	+	+	+	−	−	−
52	Eschweilenol A or isomer	C_20_H_10_O_11_	−	+	−	−	−	−
53	Dimethoxy-trihydroxy(iso)flavone-O-hexoside	C_23_H_24_O_12_	−	−	−	−	+	+
54	Dihydroactinidiolide	C_11_H_16_O_2_	−	−	−	+	−	−
55	Trihydroxy-trimethoxy(iso)flavone-O-hexoside isomer 2	C_24_H_26_O_13_	−	−	−	−	+	+
56 ^1^	Quercetin (3,3′,4′,5,7-Pentahydroxyflavone)	C_15_H_10_O_7_	+	+	+	+	+	+
57 ^1^	Luteolin (3′,4′,5,7-Tetrahydroxyflavone)	C_15_H_10_O_6_	−	−	−	+	+	−
58	3,3′-Di-O-methylellagic acid	C_16_H_10_O_8_	+	+	+	−	−	−
59	Methoxy-tetrahydroxy(iso)flavone	C_16_H_12_O_7_	+	+	+	−	−	−
60	Methoxy-tetrahydroxy(iso)flavone isomer 1	C_16_H_12_O_7_	−	−	−	+	+	+
61 ^1^	Kaempferol (3,4′,5,7-Tetrahydroxyflavone)	C_15_H_10_O_6_	−	−	−	+	+	+
62	Methoxy-tetrahydroxy(iso)flavone isomer 2	C_16_H_12_O_7_	−	−	−	+	+	+
63 ^1^	Tricin (3′,5′-Dimethoxy-4′,5,7-trihydroxyflavone)	C_17_H_14_O_7_	+	+	+	+	+	+
64	Salcolin A (Tricin-4′-O-(erythro-β-guaiacylglyceryl)ether)	C_27_H_26_O_11_	−	−	−	−	+	−
65	Methoxy-trihydroxy(iso)flavone isomer 1	C_16_H_12_O_6_	−	−	−	+	+	−
66	Dimethoxy-tetrahydroxy(iso)flavone	C_17_H_14_O_8_	−	−	+	−	−	−
67	3,3′,4-Tri-O-methylellagic acid	C_17_H_12_O_8_	+	+	+	−	−	−
68	Methoxy-trihydroxy(iso)flavone isomer 2	C_16_H_12_O_6_	−	−	−	+	+	−
69	Dimethoxy-trihydroxy(iso)flavone isomer 1	C_17_H_14_O_7_	+	−	−	−	−	−
70	Salcolin B (Tricin-4′-O-(threo-β-guaiacylglyceryl)ether)	C_27_H_26_O_11_	−	−	−	−	+	−
71	3,3′,4,4′-Tetra-O-methylellagic acid	C_18_H_14_O_8_	+	+	+	+	+	+
72	Dimethoxy-trihydroxy(iso)flavone isomer 2	C_17_H_14_O_7_	+	−	−	−	−	−
73	Dihydroxy-dimethoxy(iso)flavone	C_17_H_14_O_6_	−	−	−	+	+	−
74	Hydroxy-tetramethoxy(iso)flavone isomer 1	C_19_H_18_O_7_	−	−	−	+	+	+
75	Hydroxy-tetramethoxy(iso)flavone isomer 2	C_19_H_18_O_7_	−	−	−	+	+	+

^1^ Confirmed by standard. +: present; −: absent.

**Table 3:** Antioxidant properties of the tested extracts.

Parts	Solvents	DPPH	ABTS	CUPRAC	FRAP	Phosphomolybdenum	Metal Chelating
		(mg TE/g)				(mmol TE/g)	(mg EDTAE/g)
Leaves	EA	10.71 ± 0.78 ^f^	2.97 ± 0.66 ^f^	69.51 ± 0.90 ^f^	30.97 ± 0.66 ^f^	2.61 ± 0.11 ^c^	19.57 ± 0.50 ^b^
	MeOH	197.38 ± 0.51 ^b^	345.15 ± 4.27 ^b^	395.81 ± 12.02 ^b^	256.72 ± 3.39 ^b^	3.17 ± 0.07 ^b^	12.41 ± 1.28 ^d^
	Water	146.91 ± 1.77 ^c^	247.75 ± 3.05 ^c^	328.08 ± 3.79 ^c^	204.92 ± 1.91 ^c^	2.24 ± 0.07 ^cd^	17.83 ± 0.17 ^b^
Stem bark	EA	31.68 ± 0.46 ^e^	22.98 ± 0.93 ^e^	81.54 ± 1.30 ^e^	38.57 ± 0.37 ^e^	2.03 ± 0.15 ^d^	26.59 ± 0.25 ^a^
	MeOH	491.59 ± 0.37 ^a^	804.22 ± 5.03 ^a^	1066.93 ± 12.02 ^a^	633.44 ± 10.13 ^a^	7.11 ± 0.39 ^a^	8.70 ± 0.33 ^e^
	Water	95.26 ± 1.41 ^d^	118.34 ± 4.38 ^d^	241.37 ± 0.78 ^d^	136.26 ± 0.40 ^d^	6.92 ± 0.20 ^a^	14.44 ± 0.94 ^c^

Values are reported as mean ± S.D. EA: ethyl acetate; MeOH: methanolic; TE: trolox equivalent; EDTAE: EDTA equivalent. Different letters (a–f) indicate significant differences in the tested extracts (*p* < 0.05).

**Table 4:** Enzyme inhibitory properties of the tested extracts.

Parts	Solvents	AChE	BChE	Tyrosinase	Amylase	Glucosidase
		(mg GALAE/g)		(mg KAE/g)	(mmol ACAE/g)	
Leaves	EA	2.45 ± 0.30 ^d^	6.21 ± 0.17 ^c^	103.13 ± 1.23 ^d^	0.94 ± 0.01 ^c^	6.24 ± 0.29 ^b^
	MeOH	4.64 ± 0.08 ^b^	9.27 ± 1.08 ^b^	150.71 ± 0.57 ^b^	1.38 ± 0.03 ^a^	na
	Water	2.39 ± 0.05 ^d^	3.15 ± 0.54 ^d^	46.04 ± 3.47 ^e^	0.21 ± 0.02 ^d^	na
Stem bark	EA	4.37 ± 0.17 ^b^	7.70 ± 1.54 ^bc^	106.70 ± 1.41 ^d^	0.91 ± 0.04 ^c^	6.68 ± 0.01 ^a^
	MeOH	5.18 ± 0.04 ^a^	12.79 ± 0.93 ^a^	157.07 ± 0.37 ^a^	1.35 ± 0.05 ^a^	na
	Water	3.36 ± 0.10 ^c^	6.36 ± 0.03 ^c^	123.23 ± 1.15 ^c^	1.06 ± 0.04 ^b^	na

Values are reported as mean ± S.D. EA: Ethyl acetate; MeOH: methanolic; GALAE: Galantamine equivalent; KAE: Kojic acid equivalent; ACAE: Acarbose equivalent. na: not active. Different letters (a–e) indicate significant differences in the tested extracts (*p* < 0.05).

**Table 5:** Minimal inhibitory concentrations (MICs) of *B. ferruginea* methanolic extract against selected bacterial strains.

Bacteria (ID Strain)	Minimum Inhibitory Concentration (MIC) * µg/mL
**Gram−**	
*Escherichia coli* (ATCC 10536)	2.48 (1.562–3.125)
*Escherichia coli* (PeruMycA 2)	2.48 (1.562–3.125)
*Escherichia coli* (PeruMycA 3)	62.99 (50–100)
*Pseudomonas aeruginosa* (PeruMyc 5)	>200
*Salmonella typhy* (PeruMyc 7)	125 (100–200)
**Gram+**	
*Bacillus cereus* (PeruMycA 4)	39.68 (25–50)
*Bacillus subtilis* (PeruMyc 6)	31.50 (25–50)
*Staphylococcus aureus* (ATCC 6538)	62.99 (50–100)

* MIC values are reported as geometric means of three independent replicates (*n* = 3); MIC ranges are reported within brackets. MIC values are reported as < [lowest concentration tested].

**Table 6:** Minimal inhibitory concentrations (MICs) of *B. ferruginea* methanolic extract against selected dermatophytes and yeasts.

Dermatophytes (ID Strain)	Minimum Inhibitory Concentration (MIC) * µg/mL
*Trichophyton mentagrophytes* (CCF 4823)	31.49 (25–50)
*Trichophyton tonsurans* (4834)	19.84 (12.5–25)
*Trichophyton rubrum* (4933)	9.92 (6.25–12.5)
*Arthroderma quadrifidum* (5792)	4.96 (3.125–6.25)
*Tricophyton mentagrophytes* (5930)	19.84 (12.5–25)
*Nannizia gypseum* (6261)	9.92 (6.25–12.5)
*Arthroderma currei* (5207)	9.92 (6.25–12.5)
*Arthroderma insingulare* (5417)	7.87 (6.25–12.5)
**Yeasts (ID Strain)**	
*Candida tropicalis* (6148)	62.99 (50–100)
*Candida albicans* (6379)	62.99 (50–100)
*Candida parapsilosis* (6551)	31.49 (25–50)
*Candida albicans* (6183)	62.99 (50–100)

* MIC values are reported as geometric means of three independent replicates (*n* = 3); MIC ranges are reported within brackets. MIC values are reported as < [lowest concentration tested].

## Data Availability

The data presented in this study are available on request from the corresponding author.

## Supplementary Materials

The following are available online at https://www.mdpi.com/2079-6382/10/2/223/s1: Figure S1: Extracted Ion Chromatograms of Ellagic acid and its derivatives (XIC), Figure S2: MS2 spectrum of Ellagic acid *m*/*z* 300.99845 (ESI−), Figure S3: MS2 spectrum of Ellagic acid-O-hexoside *m*/*z* 463.05127 (ESI−), Figure S4: MS2 spectrum of Ellagic acid-O-pentoside *m*/*z* 433.04071 (ESI−), Figure S5: MS2 spectrum of Eschweilenol C (Ellagic acid-4-O-rhamnoside) *m*/*z* 447.05636 (ESI−), Figure S6: MS2 spectrum of Ducheside A (3-O-Methylellagic acid-4′-O-xyloside) *m*/*z* 447.05636 (ESI−), Figure S7: MS2 spectrum of Eschweilenol A or isomer *m*/*z* 425.01449 (ESI−), Table S1: Chemical composition of ethyl acetate extract from stem barks, Table S2: Chemical composition of methanol extract from stem barks, Table S3: Chemical composition of water extract from stem barks, Table S4: Chemical composition of ethyl acetate extract from leaves, Table S5: Chemical composition of methanol extract from leaves, Table S6: Chemical composition of water extract from leaves.
