# Supplementary material for: Pharmacological Potential and Chemical Characterization of Bridelia ferruginea Benth.—A Native Tropical African Medicinal Plant
Source: Antibiotics (Basel). 2021 Feb 23;10(2):223. doi: 10.3390/antibiotics10020223 (PMC7926895; doi:10.3390/antibiotics10020223)
Supplement: Supplementary file 1 [file antibiotics-10-00223-s001.pdf]

Article

Pharmacological Potential and Chemical Characterization of *Bridelia ferruginea* Benth.—A Native Tropical African Medicinal Plant

Mohamad Fawzi Mahomoodally [1,2,*], Sharmeen Jugreet [3], Kouadio Ibrahime Sinan [4], Gokhan Zengin [4], Gunes Ak [4], Ramazan Ceylan [4], József Jekő [5], Zoltán Cziáky [5], Paola Angelini [6,*], Giancarlo Angeles Flores [6], Roberto Venanzoni [6], Simonetta Cristina Di Simone [7], Luigi Menghini [7], Giustino Orlando [7], Claudio Ferrante [7], Ouattara Katinan Etienne [8] and Massimo Tacchini [9]

[1] Department for Management of Science and Technology Development, Ton Duc Thang University, Ho Chi Minh City 758307, Vietnam
[2] Faculty of Applied Sciences, Ton Duc Thang University, Ho Chi Minh City 758307, Vietnam
[3] Department of Health Sciences, Faculty of Medicine and Health Sciences, University of Mauritius, Réduit 80837, Mauritius; bibi.jugreet2@umail.uom.ac.mu
[4] Physiology and Biochemistry Research Laboratory, Department of Biology, Science Faculty, Selcuk University, Konya 42130, Turkey; sinankouadio@gmail.com (K.I.S.); gokhanzengin@selcuk.edu.tr (G.Z.); akguneselcuk@gmail.com (G.A.); biyoram7@gmail.com (R.C.)
[5] Agricultural and Molecular Research and Service Institute, University of Nyíregyháza, 4400 Nyíregyháza, Hungary jjozsi@gmail.com (J.J.); cziaky.zoltan@nye.hu (Z.C.)
[6] Department of Chemistry, Biology and Biotechnology, University of Perugia, 06100 Perugia, Italy; giancarlo.angelesflores@studenti.unipg.it (G.A.F.); roberto.venanzoni@unipg.it (R.V.)
[7] Department of Pharmacy, Università degli Studi "Gabriele d'Annunzio", via dei Vestini 31, 66100 Chieti, Italy; disimonesimonetta@gmail.com (S.C.D.S.); luigi.menghini@unich.it (L.M.); giustino.orlando@unich.it (G.O.); claudio.ferrante@unich.it (C.F.)
[8] Laboratoire de Botanique, UFR Biosciences, Université Félix Houphouët-Boigny, 01 Abidjan, Cote D'Ivoire, katinan.etienne@gmail.com
[9] Department of Life Sciences and Biotechnology (SVeB), UR7 Terra&Acqua Tech, University of Ferrara, 44121 Ferrara, Italy; massimo.tacchini@unife.it
* Correspondence: mohamad.fawzi.mahomoodally@tdtu.edu.vn (M.F.M); paola.angelini@unipg.it (P.A.)

Table S1. Chemical composition of ethyl acetate extract from stem barks

No.	Name	Formula	Rt	[M + H]+	[M - H]-	Fragment 1	Fragment 2	Fragment 3	Fragment 4	Fragment 5	Literature
1[1]	Gallic acid (3,4,5-Trihydroxybenzoic acid)	C7H6O5	2,63		169,01370	125,0229	97,0281	81,0331	79,0174	69,0329	
2	Gallocatechin	C15H14O7	5,53		305,06613	261,0764	219,0662	167,0341	137,0231	125,0229	
3	Pantothenic acid	C9H17NO5	6,08	220,11850		202,1071	184,0969	174,1127	116,0346	90,0553	
4[1]	Epigallocatechin	C15H14O7	14,17		305,06613	261,0772	219,0654	167,0337	137,0231	125,0229	
5[1]	Epigallocatechin-3-O-gallate (Teatannin II)	C22H18O11	16,81		457,07709	331,0462	305,0656	169,0130	161,0232	125,0229	
6[1]	Epicatechin	C15H14O6	17,53		289,07121	245,0811	203,0704	151,0385	125,0228	109,0279	
7	Corilagin	C27H22O18	17,91		633,07279	463,0516	419,0624	300,9989	275,0198	169,0132	[2]
8[1]	Ferulic acid	C10H10O4	19,85		193,05009	178,0260	149,0595	137,0231	134,0360	121,0279	
9	Ellagic acid-O-hexoside	C20H16O13	20,34		463,05127	300,9988	299,9910	298,9852	283,9948		
10	Methylellagic acid-O-hexoside	C21H18O13	22,26		477,06692	315,0147	314,0071	299,9911	298,9833	270,9886	
11	Isovitexin (Apigenin-6-C-glucoside)	C21H20O10	22,70	433,11348		415,1014	397,0918	337,0702	313,0703	283,0597	
12	Ellagic acid-O-pentoside	C19H14O12	23,23		433,04071	300,9989	299,9910	298,9810	257,0085		
13	Eschweilenol C (Ellagic acid-4-O-rhamnoside)	C20H16O12	23,56		447,05636	300,9988	299,9909	298,9851			
14	Ellagic acid	C14H6O8	23,92		300,99845	283,9963	257,0089	229,0135	201,0184	185,0234	
15	Ducheside A (3-O-Methylellagic acid-4'-O-xyloside)	C20H16O12	25,25		447,05636	315,0146	314,0068	299,9910	298,9831	270,9885	
16	Dimethoxy-tetrahydroxy(iso)flavone-O-hexoside	C23H24O13	25,45		507,11387	345,0607	344,0534	329,0309	301,0348	273,0411	
17	3-O-Methylellagic acid-O-rhamnoside	C21H18O12	25,78		461,07201	315,0147	299,9911	298,9843	270,9878		
18	3-O-Methylellagic acid	C15H8O8	26,23		315,01410	299,9908	244,0011	228,0065	200,0102		
19[1]	Quercetin (3,3',4',5,7-Pentahydroxyflavone)	C15H10O7	27,49		301,03483	273,0405	178,9977	151,0024	121,0280	107,0121	
20	3,3'-Di-O-methylellagic acid	C16H10O8	28,42		329,02975	314,0067	298,9832	270,9885			
21	Methoxy-tetrahydroxy(iso)flavone	C16H12O7	28,74		315,05048	300,0274	271,0245	255,0295	243,0294		
22[1]	Tricin (3',5'-Dimethoxy-4',5,7-trihydroxyflavone)	C17H14O7	30,37		329,06613	314,0432	313,0357	299,0196	271,0249	227,0337	
23	3,3',4-Tri-O-methylellagic acid	C17H12O8	30,79		343,04540	328,0224	312,9991	297,9751	285,0039		
24	Dimethoxy-trihydroxy(iso)flavone isomer 1	C17H14O7	31,05		329,06613	314,0433	299,0197	285,0401	271,0248	243,0293	
25	3,3',4,4'-Tetra-O-methylellagic acid	C18H14O8	32,62	359,07670		344,0522	343,0444	329,0288	313,0337		
26	Dimethoxy-trihydroxy(iso)flavone isomer 2	C17H14O7	33,27		329,06613	314,0433	299,0197	271,0248	243,0294		

Table S2. Chemical composition of methanol extract from stem barks

No.	Name	Formula	Rt	$[M + H]^+$	$[M - H]^-$	Fragment 1	Fragment 2	Fragment 3	Fragment 4	Fragment 5	Literature
1	Epigallocatechin-(7-O-4')-gallocatechin or isomer	C30H26O13	1,27		593,12952	467,0979	423,0721	407,0772	305,0667	125,0229	[69]
2[1]	Gallic acid (3,4,5-Trihydroxybenzoic acid)	C7H6O5	2,62		169,01370	125,0229	97,0280	81,0332	79,0174	69,0330	
3	Gallocatechin	C15H14O7	5,51		305,06613	261,0764	219,0654	167,0337	137,0230	125,0229	
4	Pantothenic acid	C9H17NO5	6,04	220,11850		202,1074	184,0969	174,1127	116,0345	90,0554	
5	Epigallocatechin-(7-O-4')-gallocatechin or isomer	C30H26O13	6,16		593,12952	467,0988	441,0814	423,0721	305,0667	125,0229	[69]
6	Prodelphinidin C	C30H26O13	11,65		593,12952	425,0880	407,0771	289,0718	177,0181	125,0229	
7	Epigallocatechin-(7-O-4')-gallocatechin or isomer	C30H26O13	13,20		593,12952	467,0985	441,0837	423,0720	305,0668	125,0229	[69]
8[1]	Catechin	C15H14O6	13,96		289,07121	245,0815	203,0706	151,0386	125,0230	109,0280	
9[1]	Epigallocatechin	C15H14O7	14,17		305,06613	261,0766	219,0656	167,0338	137,0231	125,0230	
10	Epigallocatechin-(7-O-4')-gallocatechin or isomer	C30H26O13	14,76		593,12952	467,0984	441,0831	423,0721	305,0667	125,0229	[69]
11[1]	Epigallocatechin-3-O-gallate (Teatannin II)	C22H18O11	16,82		457,07709	331,0462	305,0668	169,0130	161,0232	125,0229	
12	Dihydrokaempferol-O-hexoside	C21H22O11	17,51		449,10839	287,0574	269,0457	259,0610	125,0228		
13[1]	Epicatechin	C15H14O6	17,53		289,07121	245,0815	203,0706	151,0388	125,0229	109,0280	
14	Corilagin	C27H22O18	17,91		633,07279	463,0524	419,0587	300,9990	275,0197	169,0131	[2]
15[1]	Epicatechin-3-O-gallate	C22H18O10	19,79		441,08218	289,0719	245,0817	169,0131	125,0230	109,0279	
16[1]	Ferulic acid	C10H10O4	19,86		193,05009	178,0260	149,0594	137,0230	134,0360	121,0278	
17	Ellagic acid-O-hexoside	C20H16O13	20,34		463,05127	300,9990	299,9911	298,9852	283,9989		
18	Methylellagic acid-O-hexoside	C21H18O13	22,42		477,06692	315,0146	300,9983	299,9910	298,9832	270,9881	
19	Myricitrin (Myricetin-3-O-rhamnoside)	C21H20O12	22,46		463,08765	317,0308	316,0223	287,0197	271,0246	151,0024	[2]
20	Isovitexin (Apigenin-6-C-glucoside)	C21H20O10	22,70	433,11348		415,1008	397,0908	337,0700	313,0705	283,0598	
21	Coatline A or isomer	C21H24O10	23,06		435,12913	417,1211	345,0979	315,0875	221,0452	209,0449	
22	Ellagic acid-O-pentoside	C19H14O12	23,27		433,04071	300,9988	299,9911	298,9810	257,0080		
23	Eschweilenol C (Ellagic acid-4-O-rhamnoside)	C20H16O12	23,58		447,05636	300,9988	299,9910				
24	Ellagic acid	C14H6O8	23,94		300,99845	283,9966	257,0087	229,0135	201,0185	185,0235	
25[1]	Myricetin (3,3',4',5,5',7-Hexahydroxyflavone)	C15H10O8	24,70		317,02974	271,0248	192,0061	178,9975	151,0023	137,0231	[70]
26	Tricin-O-hexoside	C23H24O12	24,74		491,11896	476,0949	461,0718	329,0655	313,0356	299,0212	
27	Ducheside A (3-O-Methylellagic acid-4'-O-xyloside)	C20H16O12	25,26		447,05636	315,0146	314,0070	299,9910	298,9833	270,9885	
28	Dimethoxy-tetrahydroxy(iso)flavone-O-hexoside	C23H24O13	25,45		507,11387	345,0606	344,0539	329,0306	301,0351	273,0399	

29	3-O-Methylellagic acid-O-rhamnoside	C21H18O12	25,80		461,07201	315,0146	299,9910	298,9829	270,9884	
30	Dihydroxy(iso)flavone-C-hexoside	C21H20O9	26,15	417,11856		399,1057	381,0956	321,0758	297,0752	267,0651
31	3-O-Methylellagic acid	C15H8O8	26,24		315,01410	299,9910	244,0014	228,0058	200,0111	
32	Eschweilenol A or isomer	C20H10O11	26,39		425,01449	300,9988	299,9909	298,9835		
33[1]	Quercetin (3,3',4',5,7-Pentahydroxyflavone)	C15H10O7	27,50		301,03483	273,0405	178,9972	151,0023	121,0279	107,0123
34	3,3'-Di-O-methylellagic acid	C16H10O8	28,42		329,02975	314,0069	298,9832	270,9885		
35	Methoxy-tetrahydroxy(iso)flavone	C16H12O7	28,73		315,05048	300,0273	271,0245	255,0296	243,0297	
36[1]	Tricin (3',5'-Dimethoxy-4',5,7-trihydroxyflavone)	C17H14O7	30,38		329,06613	314,0433	313,0356	299,0196	271,0245	227,0335
37	3,3',4-Tri-O-methylellagic acid	C17H12O8	30,78		343,04540	328,0223	312,9990	297,9757	285,0043	
38	3,3',4,4'-Tetra-O-methylellagic acid	C18H14O8	32,63	359,07670		344,0523	343,0445	329,0288	313,0342	

Table S3. Chemical composition of water extract from stem barks

No.	Name	Formula	Rt	$[M + H]^+$	$[M - H]^-$	Fragment 1	Fragment 2	Fragment 3	Fragment 4	Fragment 5	Literature
1[1]	Gallic acid (3,4,5-Trihydroxybenzoic acid)	C7H6O5	2,62		169,01370	125,0230	97,0280	81,0331	79,0174	69,0330	
2	Gallocatechin	C15H14O7	5,53		305,06613	261,0771	219,0657	167,0339	137,0232	125,0230	
3	Pantothenic acid	C9H17NO5	6,09	220,11850		202,1076	184,0972	174,1125	116,0345	90,0555	
4	Epigallocatechin-(7-O-4')-gallocatechin or isomer	C30H26O13	6,23		593,12952	467,0985	441,0833	423,0723	305,0671	125,0231	[69]
5	Prodelphinidin C	C30H26O13	11,69		593,12952	425,0883	407,0776	289,0721	177,0183	125,0230	
6	Epigallocatechin-(7-O-4')-gallocatechin or isomer	C30H26O13	13,23		593,12952	467,0985	441,0830	423,0724	305,0670	125,0230	[69]
7[1]	Catechin	C15H14O6	13,97		289,07121	245,0818	203,0707	151,0389	125,0230	109,0281	
8[1]	Epigallocatechin	C15H14O7	14,20		305,06613	261,0766	219,0660	167,0338	137,0232	125,0230	
9	Epigallocatechin-(7-O-4')-gallocatechin or isomer	C30H26O13	14,76		593,12952	467,0985	441,0824	423,0720	305,0672	125,0230	[69]
10[1]	Epigallocatechin-3-O-gallate (Teatannin II)	C22H18O11	16,83		457,07709	331,0472	305,0669	169,0132	161,0233	125,0230	
11	5-O-(4-Coumaroyl)quinic acid	C16H18O8	17,39		337,09235	191,0553	173,0444	163,0389	119,0488	93,0330	
12	Dihydrokaempferol-O-hexoside	C21H22O11	17,52		449,10839	287,0565	269,0457	259,0612	125,0230		
13[1]	Epicatechin	C15H14O6	17,54		289,07121	245,0817	203,0709	151,0389	125,0230	109,0281	
14	Corilagin	C27H22O18	17,92		633,07279	463,0523	419,0622	300,9992	275,0200	169,0129	[2]
15	5-O-Feruloylquinic acid	C17H20O9	18,43		367,10291	193,0501	191,0555	173,0446	134,0360	93,0331	
16[1]	Epicatechin-3-O-gallate	C22H18O10	19,80		441,08218	289,0722	245,0817	169,0132	125,0230	109,0280	
17[1]	Ferulic acid	C10H10O4	19,88		193,05009	178,0262	149,0600	137,0232	134,0361	121,0282	
18	Ellagic acid-O-hexoside	C20H16O13	20,36		463,05127	300,9992	299,9914	298,9855	283,9965		
19[1]	Vitexin (Apigenin-8-C-glucoside)	C21H20O10	21,82	433,11348		415,1031	397,0927	337,0690	313,0708	283,0602	
20	Methylellagic acid-O-hexoside	C21H18O13	22,46		477,06692	315,0150	300,9978	299,9913	298,9836	270,9887	
21	Myricitrin (Myricetin-3-O-rhamnoside)	C21H20O12	22,50		463,08765	317,0293	316,0226	287,0206	271,0256	151,0026	[2]
22	Isovitexin (Apigenin-6-C-glucoside)	C21H20O10	22,73	433,11348		415,1020	397,0922	337,0705	313,0708	283,0603	
23	Coatline A or isomer	C21H24O10	23,10		435,12913	417,1182	345,0979	315,0878	221,0447	209,0452	
24	Ellagic acid-O-pentoside	C19H14O12	23,28		433,04071	300,9992	299,9914	298,9836	257,0094		
25	Eschweilenol C (Ellagic acid-4-O-rhamnoside)	C20H16O12	23,62		447,05636	300,9992	299,9913				
26	Ellagic acid	C14H6O8	23,96		300,99845	283,9963	257,0092	229,0136	201,0190	185,0237	
27[1]	Myricetin (3,3',4',5,5',7-Hexahydroxyflavone)	C15H10O8	24,74		317,02974	271,0254	192,0060	178,9977	151,0025	137,0233	[70]
28	Ducheside A (3-O-Methylellagic acid-4'-O-xyloside)	C20H16O12	25,30		447,05636	315,0151	314,0071	299,9914	298,9837	270,9885	
29	Dimethoxy-tetrahydroxy(iso)flavone-O-hexoside	C23H24O13	25,49		507,11387	345,0621	344,0540	329,0305	301,0356	273,0411	

30	3-O-Methylellagic acid-O-rhamnoside	C21H18O12	25,84		461,07201	315,0150	299,9913	298,9835	270,9892	
31	Dihydroxy(iso)flavone-C-hexoside	C21H20O9	26,19	417,11856		399,1073	381,0970	321,0760	297,0759	267,0652
32	3-O-Methylellagic acid	C15H8O8	26,28		315,01410	299,9913	244,0006	228,0058	200,0100	
33[1]	Quercetin (3,3',4',5,7-Pentahydroxyflavone)	C15H10O7	27,53		301,03483	273,0402	178,9979	151,0025	121,0281	107,0125
34	3,3'-Di-O-methylellagic acid	C16H10O8	28,47		329,02975	314,0072	298,9835	270,9886		
35	Methoxy-tetrahydroxy(iso)flavone	C16H12O7	28,77		315,05048	300,0270	271,0250	255,0298	243,0292	
36[1]	Tricin (3',5'-Dimethoxy-4',5,7-trihydroxyflavone)	C17H14O7	30,42		329,06613	314,0436	313,0355	299,0200	271,0249	227,0345
37	Dimethoxy-tetrahydroxy(iso)flavone	C17H14O8	30,53		345,06104	330,0386	329,0303	315,0151	287,0204	
38	3,3',4-Tri-O-methylellagic acid	C17H12O8	30,83		343,04540	328,0227	312,9993	297,9756	285,0042	
39	3,3',4,4'-Tetra-O-methylellagic acid	C18H14O8	32,68	359,07670		344,0527	343,0450	329,0293	313,0345	

Table S4. Chemical composition of ethyl acetate extract from leaves

No.	Name	Formula	Rt	[M + H]+	[M - H]-	Fragment 1	Fragment 2	Fragment 3	Fragment 4	Fragment 5	Literature
1[1]	Chlorogenic acid (3-O-Caffeoylquinic acid)	C16H18O9	14,85	355,10291		163,0387	145,0283	135,0441	117,0336	89,0387	
2	Myricetin-O-hexoside	C21H20O13	21,42		479,08257	317,0302	316,0223	287,0197	271,0248	178,9977	
3[1]	Vitexin (Apigenin-8-C-glucoside)	C21H20O10	21,85	433,11348		415,1023	397,0911	337,0700	313,0702	283,0597	
4	Isovitexin (Apigenin-6-C-glucoside)	C21H20O10	22,75	433,11348		415,1018	397,0912	337,0702	313,0702	283,0597	
5[1]	Rutin (Quercetin-3-O-rutinoside)	C27H30O16	23,52	611,16122		465,0982	303,0494	129,0548	85,0288	71,0497	
6	Tricin-O-hexoside isomer 1	C23H24O12	24,76		491,11896	476,0953	461,0721	329,0660	313,0353	299,0196	
7[1]	Quercitrin (Quercetin-3-O-rhamnoside)	C21H20O11	24,98		447,09274	301,0353	300,0275	271,0248	255,0297	151,0024	
8	Kaempferol-3-O-rutinoside (Nicotiflorin)	C27H30O15	25,35		593,15065	327,0518	285,0403	284,0326	255,0295	227,0341	
9	Dimethoxy-tetrahydroxy(iso)flavone-O-hexoside	C23H24O13	25,48		507,11387	345,0614	344,0535	329,0302	301,0354	273,0405	
10	Tricin-O-hexoside isomer 2	C23H24O12	25,59		491,11896	476,0961	461,0747	329,0664	313,0355	299,0198	
11	Trihydroxy-trimethoxy(iso)flavone-O-hexoside isomer 1	C24H26O13	25,63		521,12952	506,1068	491,0844	359,0764	343,0457	329,0295	
12	Isorhamnetin-3-O-rutinoside (Narcissin)	C28H32O16	25,71		623,16122	315,0510	314,0433	300,0275	299,0196	271,0247	
13	Dihydroactinidiolide	C11H16O2	27,06	181,12286		163,1115	145,1011	135,1168	121,1012	107,0858	
14[1]	Quercetin (3,3',4',5,7-Pentahydroxyflavone)	C15H10O7	27,50		301,03483	273,0412	178,9975	151,0023	121,0280	107,0123	
15[1]	Luteolin (3',4',5,7-Tetrahydroxyflavone)	C15H10O6	28,38		285,03991	217,0494	199,0400	175,0386	151,0024	133,0282	
16	Methoxy-tetrahydroxy(iso)flavone isomer 1	C16H12O7	28,74		315,05048	300,0274	271,0247	255,0297	243,0291		
17[1]	Kaempferol (3,4',5,7-Tetrahydroxyflavone)	C15H10O6	29,86		285,03991	257,0449	229,0502	185,0606	151,0022	107,0122	
18	Methoxy-tetrahydroxy(iso)flavone isomer 2	C16H12O7	30,36		315,05048	300,0274	271,0247	243,0290			
19[1]	Tricin (3',5'-Dimethoxy-4',5,7-trihydroxyflavone)	C17H14O7	30,39		329,06613	314,0433	313,0347	299,0197	271,0247	227,0343	
20	Methoxy-trihydroxy(iso)flavone isomer 1	C16H12O6	30,44	301,07121		286,0468	258,0517				
21	Methoxy-trihydroxy(iso)flavone isomer 2	C16H12O6	30,89	301,07121		286,0469	285,0392	258,0526			
22	3,3',4,4'-Tetra-O-methylellagic acid	C18H14O8	32,62	359,07670		344,0522	343,0442	329,0288	313,0341		
23	Dihydroxy-dimethoxy(iso)flavone	C17H14O6	35,48	315,08687		300,0623	299,0544	272,0678	271,0597	257,0437	
24	Hydroxy-tetramethoxy(iso)flavone isomer 1	C19H18O7	36,70	359,11308		344,0879	343,0806	329,0651	313,0704	298,0831	
25	Hydroxy-tetramethoxy(iso)flavone isomer 2	C19H18O7	37,04	359,11308		344,0885	343,0806	329,0651	315,0852	301,0702	

Table S5. Chemical composition of methanol extract from leaves

No.	Name	Formula	Rt	[M + H]+	[M - H]-	Fragment 1	Fragment 2	Fragment 3	Fragment 4	Fragment 5	Literature
1[1]	Gallic acid (3,4,5-Trihydroxybenzoic acid)	C7H6O5	2,64		169,01370	125,0229	97,0280	81,0332	79,0174	69,0330	
2	Gallocatechin	C15H14O7	5,57		305,06613	261,0770	219,0656	167,0337	137,0231	125,0229	
3	Pantothenic acid	C9H17NO5	6,05	220,11850		202,1073	184,0968	174,1120	116,0343	90,0554	
4	Kynurenic acid	C10H7NO3	13,82	190,05042		162,0548	144,0437	116,0497	89,0388		
5[1]	Catechin	C15H14O6	14,00		289,07121	245,0816	203,0707	151,0389	125,0229	109,0280	
6[1]	Epigallocatechin	C15H14O7	14,22		305,06613	261,0760	219,0655	167,0337	137,0231	125,0229	
7[1]	Chlorogenic acid (3-O-Caffeoylquinic acid)	C16H18O9	14,85	355,10291		163,0387	145,0283	135,0440	117,0337	89,0389	
8[1]	Epigallocatechin-3-O-gallate (Teatannin II)	C22H18O11	16,83		457,07709	331,0462	305,0667	169,0129	161,0229	125,0228	
9[1]	Epicatechin	C15H14O6	17,56		289,07121	245,0811	203,0707	151,0384	125,0229	109,0279	
10	Myricetin-O-hexoside	C21H20O13	21,41		479,08257	317,0300	316,0222	287,0197	271,0247	178,9973	
11[1]	Vitexin (Apigenin-8-C-glucoside)	C21H20O10	21,82	433,11348		415,1020	397,0915	337,0704	313,0703	283,0599	
12	Myricitrin (Myricetin-3-O-rhamnoside)	C21H20O12	22,49		463,08765	317,0301	316,0222	287,0197	271,0246	151,0022	[2]
13	Isovitexin (Apigenin-6-C-glucoside)	C21H20O10	22,74	433,11348		415,1015	397,0913	337,0699	313,0702	283,0597	
14	Coatline A or isomer	C21H24O10	23,08		435,12913	417,1188	345,0978	315,0875	221,0444	209,0449	
15	Hexahydroxy(iso)flavanone	C15H12O8	23,11		319,04540	301,0351	215,0341	193,0133	153,0179	125,0229	
16[1]	Rutin (Quercetin-3-O-rutinoside)	C27H30O16	23,51	611,16122		465,1004	303,0495	129,0548	85,0289	71,0497	
17	Ellagic acid	C14H6O8	23,96		300,99845	283,9972	257,0090	229,0136	201,0184	185,0235	
18	Methoxy-pentahydroxy(iso)flavone-O-rhamnosylhexoside isomer 1	C28H32O17	24,11		639,15613	331,0460	330,0380	316,0220	315,0147	287,0198	
19	Quercetin-O-malonylhexoside	C23H22O13	24,14		549,08805	505,0988	301,0352	300,0274	271,0247	255,0294	
20	Methoxy-pentahydroxy(iso)flavone-O-rhamnosylhexoside isomer 2	C28H32O17	24,63		639,15613	331,0449	330,0365	316,0222	315,0149	287,0199	
21	Kaempferol-O-rhamnosylhexoside	C27H30O15	24,71		593,15065	327,0502	285,0404	284,0327	255,0295	227,0343	
22	Tricin-O-hexoside isomer 1	C23H24O12	24,74		491,11896	476,0958	461,0724	329,0662	313,0354	299,0198	
23[1]	Myricetin (3,3',4',5,5',7-Hexahydroxyflavone)	C15H10O8	24,77		317,02974	271,0258	192,0058	178,9976	151,0023	137,0231	[70]
24[1]	Quercitrin (Quercetin-3-O-rhamnoside)	C21H20O11	24,97		447,09274	301,0353	300,0274	271,0246	255,0294	151,0023	
25	N-trans-Feruloyltyramine	C18H19NO4	25,11	314,13924		177,0545	149,0596	145,0284	121,0649	117,0337	
26	Kaempferol-3-O-rutinoside (Nicotiflorin)	C27H30O15	25,34		593,15065	327,0504	285,0405	284,0328	255,0296	227,0342	

27	Dimethoxy-tetrahydroxy(iso)flavone-O-hexoside	C23H24O13	25,48		507,11387	345,0614	344,0535	329,0299	301,0354	273,0406
28	Tricin-O-hexoside isomer 2	C23H24O12	25,58		491,11896	476,0955	461,0726	329,0665	313,0353	299,0191
29	Trihydroxy-trimethoxy(iso)flavone-O-hexoside isomer 1	C24H26O13	25,63		521,12952	506,1054	491,0867	359,0771	343,0456	329,0300
30	Isorhamnetin-3-O-rutinoside (Narcissin)	C28H32O16	25,70		623,16122	315,0511	314,0433	300,0276	299,0197	271,0248
31	Dihydroxy(iso)flavone-C-hexoside	C21H20O9	26,15	417,11856		399,1056	381,0963	321,0750	297,0754	267,0647
32	Pentahydroxy(iso)flavanone	C15H12O7	26,16		303,05048	285,0406	217,0501	177,0181	175,0388	125,0229
33	Dimethoxy-trihydroxy(iso)flavone-O-hexoside	C23H24O12	26,46		491,11896	329,0666	328,0586	313,0354	285,0404	270,0166
34	Trihydroxy-trimethoxy(iso)flavone-O-hexoside isomer 2	C24H26O13	27,35		521,12952	506,1004	359,0768	343,0457	329,0300	328,0217
35[1]	Quercetin (3,3',4',5,7-Pentahydroxyflavone)	C15H10O7	27,51		301,03483	273,0412	178,9975	151,0023	121,0279	107,0124
36[1]	Luteolin (3',4',5,7-Tetrahydroxyflavone)	C15H10O6	28,39		285,03991	217,0500	199,0390	175,0391	151,0021	133,0281
37	Methoxy-tetrahydroxy(iso)flavone isomer 1	C16H12O7	28,74		315,05048	300,0275	271,0248	255,0296	243,0291	
38[1]	Kaempferol (3,4',5,7-Tetrahydroxyflavone)	C15H10O6	29,87		285,03991	257,0449	229,0505	185,0609	151,0022	107,0123
39	Methoxy-tetrahydroxy(iso)flavone isomer 2	C16H12O7	30,34		315,05048	300,0276	255,0284	243,0292		
40	Salcolin A (Tricin-4'-O-(erythro-β-guaiacylglyceryl)ether)	C27H26O11	30,37		525,13969	329,0664	314,0433	299,0196	195,0653	165,0544
41[1]	Tricin (3',5'-Dimethoxy-4',5,7-trihydroxyflavone)	C17H14O7	30,40		329,06613	314,0432	313,0359	299,0196	271,0248	227,0340
42	Methoxy-trihydroxy(iso)flavone isomer 1	C16H12O6	30,44	301,07121		286,0468	258,0521			
43	Methoxy-trihydroxy(iso)flavone isomer 2	C16H12O6	30,91	301,07121		286,0468	285,0393	258,0523		
44	Salcolin B (Tricin-4'-O-(threo-β-guaiacylglyceryl)ether)	C27H26O11	31,18		525,13969	329,0667	314,0433	299,0198	195,0658	165,0544
45	3,3',4,4'-Tetra-O-methylellagic acid	C18H14O8	32,64	359,07670		344,0523	343,0440	329,0286	313,0341	
46	Dihydroxy-dimethoxy(iso)flavone	C17H14O6	35,48	315,08687		300,0626	299,0540	272,0687	271,0600	257,0440
47	Hydroxy-tetramethoxy(iso)flavone isomer 1	C19H18O7	36,70	359,11308		344,0882	343,0808	329,0652	313,0704	298,0830
48	Hydroxy-tetramethoxy(iso)flavone isomer 2	C19H18O7	37,04	359,11308		344,0887	343,0804	329,0652	315,0851	301,0704

Table S6. Chemical composition of water extract from leaves

No.	Name	Formula	Rt	$[M + H]^+$	$[M - H]^-$	Fragment 1	Fragment 2	Fragment 3	Fragment 4	Fragment 5	Literature
1[1]	Gallic acid (3,4,5-Trihydroxybenzoic acid)	C7H6O5	2,60		169,01370	125,0229	97,0280	81,0331	79,0174	69,0329	
2	Gallocatechin	C15H14O7	5,53		305,06613	261,0770	219,0657	167,0338	137,0231	125,0229	
3	Pantothenic acid	C9H17NO5	6,11	220,11850		202,1072	184,0968	174,1124	116,0343	90,0554	
4	Kynurenic acid	C10H7NO3	13,85	190,05042		162,0548	144,0443	116,0500	89,0388		
5[1]	Catechin	C15H14O6	13,97		289,07121	245,0818	203,0706	151,0389	125,0230	109,0280	
6[1]	Epigallocatechin	C15H14O7	14,17		305,06613	261,0772	219,0656	167,0338	137,0231	125,0229	
7[1]	Chlorogenic acid (3-O-Caffeoylquinic acid)	C16H18O9	14,83	355,10291		163,0388	145,0284	135,0441	117,0337	89,0389	
8[1]	Epicatechin	C15H14O6	17,53		289,07121	245,0814	203,0705	151,0392	125,0232	109,0281	
9	Myricetin-O-hexoside	C21H20O13	21,40		479,08257	317,0300	316,0223	287,0198	271,0248	178,9974	
10[1]	Vitexin (Apigenin-8-C-glucoside)	C21H20O10	21,82	433,11348		415,1021	397,0918	337,0703	313,0702	283,0597	
11	Isovitexin (Apigenin-6-C-glucoside)	C21H20O10	22,74	433,11348		415,1023	397,0916	337,0702	313,0703	283,0598	
12	Hexahydroxy(iso)flavanone	C15H12O8	23,13		319,04540	301,0355	215,0341	193,0134	153,0181	125,0229	
13[1]	Rutin (Quercetin-3-O-rutinoside)	C27H30O16	23,50	611,16122		465,1017	303,0496	129,0549	85,0289	71,0497	
14	Ellagic acid	C14H6O8	23,95		300,99845	283,9965	257,0078	229,0138	201,0183	185,0237	
15	Methoxy-pentahydroxy(iso)flavone-O-rhamnosylhexoside isomer 1	C28H32O17	24,10		639,15613	331,0460	330,0381	316,0222	315,0148	287,0198	
16	Quercetin-O-malonylhexoside	C23H22O13	24,15		549,08805	505,0988	301,0354	300,0275	271,0248	255,0296	
17	Methoxy-pentahydroxy(iso)flavone-O-rhamnosylhexoside isomer 2	C28H32O17	24,63		639,15613	331,0453	330,0365	316,0223	315,0148	287,0205	
18	Kaempferol-O-rhamnosylhexoside	C27H30O15	24,72		593,15065	327,0498	285,0405	284,0327	255,0295	227,0343	
19	Tricin-O-hexoside isomer 1	C23H24O12	24,76		491,11896	476,0957	461,0727	329,0678	313,0354	299,0200	

20[1]	Myricetin (3,3',4',5,5',7-Hexahydroxyflavone)	C15H10O8	24,79		317,02974	271,0250	192,0053	178,9976	151,0024	137,0231	[70]
21[1]	Quercitrin (Quercetin-3-O-rhamnoside)	C21H20O11	24,97		447,09274	301,0353	300,0274	271,0247	255,0295	151,0023	
22	Kaempferol-3-O-rutinoside (Nicotiflorin)	C27H30O15	25,35		593,15065	327,0512	285,0404	284,0327	255,0295	227,0343	
23	Dimethoxy-tetrahydroxy(iso)flavone-O-hexoside	C23H24O13	25,48		507,11387	345,0613	344,0535	329,0299	301,0355	273,0406	
24	Tricin-O-hexoside isomer 2	C23H24O12	25,60		491,11896	476,0958	461,0728	329,0658	313,0353	299,0206	
25	Trihydroxy-trimethoxy(iso)flavone-O-hexoside isomer 1	C24H26O13	25,62		521,12952	506,1064	491,0847	359,0759	343,0454	329,0306	
26	Isorhamnetin-3-O-rutinoside (Narcissin)	C28H32O16	25,70		623,16122	315,0506	314,0433	300,0274	299,0197	271,0247	
27	Dihydroxy(iso)flavone-C-hexoside	C21H20O9	26,17	417,11856		399,1069	381,0958	321,0750	297,0755	267,0649	
28	Pentahydroxy(iso)flavanone	C15H12O7	26,19		303,05048	285,0404	217,0499	177,0181	175,0389	125,0229	
29	Dimethoxy-trihydroxy(iso)flavone-O-hexoside	C23H24O12	26,46		491,11896	329,0667	328,0587	313,0354	285,0405	270,0171	
30	Trihydroxy-trimethoxy(iso)flavone-O-hexoside isomer 2	C24H26O13	27,36		521,12952	506,1004	359,0773	343,0456	329,0301	328,0216	
31[1]	Quercetin (3,3',4',5,7-Pentahydroxyflavone)	C15H10O7	27,52		301,03483	273,0404	178,9975	151,0023	121,0280	107,0123	
32	Methoxy-tetrahydroxy(iso)flavone isomer 1	C16H12O7	28,75		315,05048	300,0277	271,0249	255,0295	243,0292		
33[1]	Kaempferol (3,4',5,7-Tetrahydroxyflavone)	C15H10O6	29,87		285,03991	257,0451	229,0508	185,0600	151,0022	107,0124	
34	Methoxy-tetrahydroxy(iso)flavone isomer 2	C16H12O7	30,34		315,05048	300,0277	271,0244	243,0292			
35[1]	Tricin (3',5'-Dimethoxy-4',5,7-trihydroxyflavone)	C17H14O7	30,40		329,06613	314,0432	313,0352	299,0197	271,0249	227,0344	
36	3,3',4,4'-Tetra-O-methylellagic acid	C18H14O8	32,63	359,07670		344,0517	343,0458	329,0288	313,0333		
37	Hydroxy-tetramethoxy(iso)flavone isomer 1	C19H18O7	36,70	359,11308		344,0882	343,0793	329,0649	313,0698	298,0830	
38	Hydroxy-tetramethoxy(iso)flavone isomer 2	C19H18O7	37,03	359,11308		344,0888	343,0811	329,0654	315,0848	301,0704	

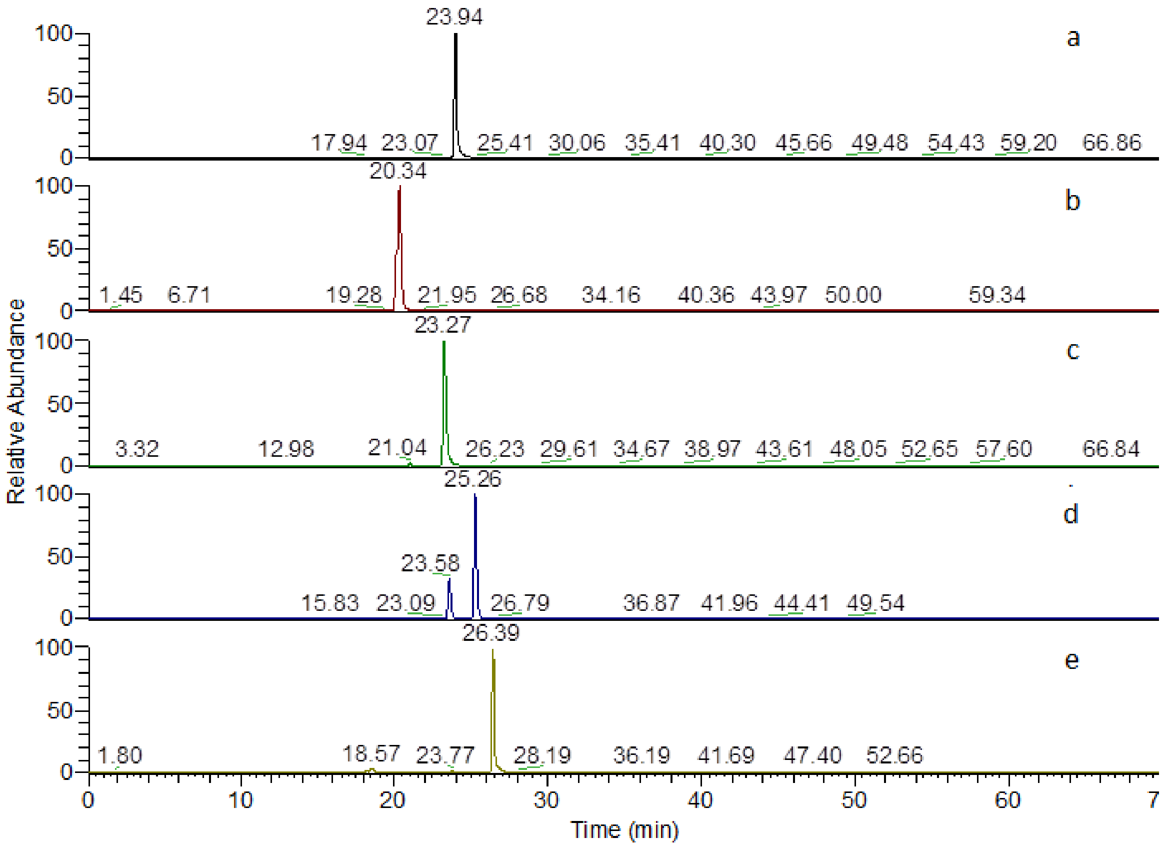

Figure S1. Extracted Ion Chromatograms of Ellagic acid and its derivatives (XIC).

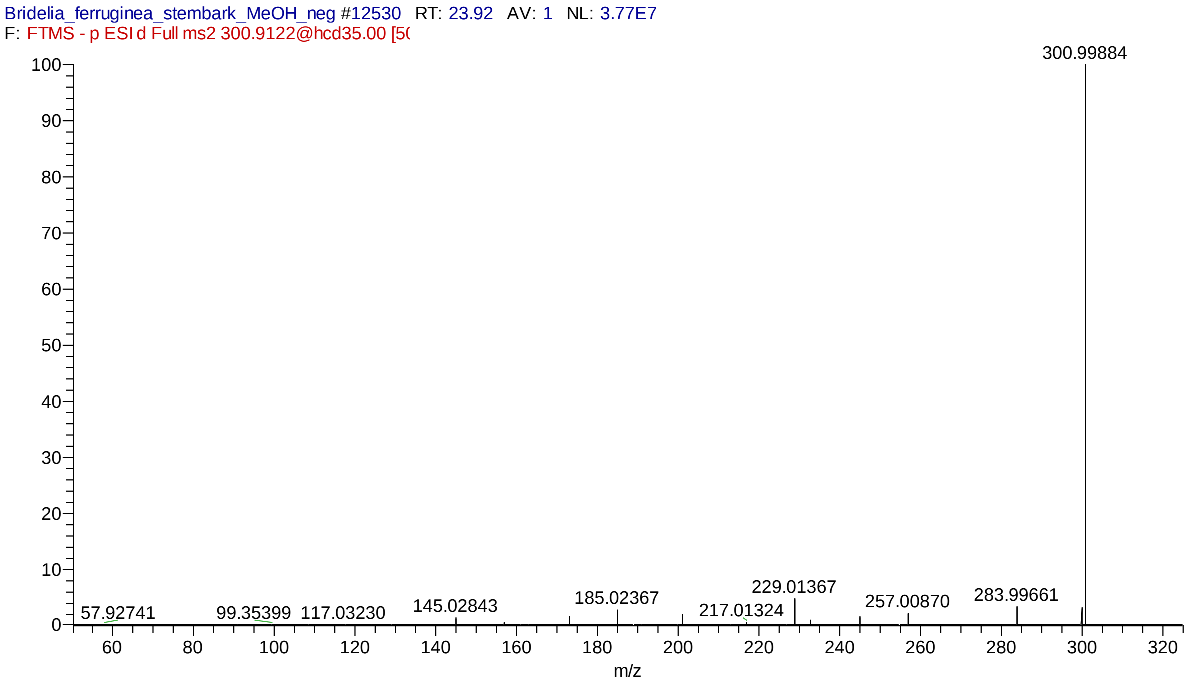

Figure S2. MS2 spectrum of Ellagic acid m/z 300.99845 (ESI-)

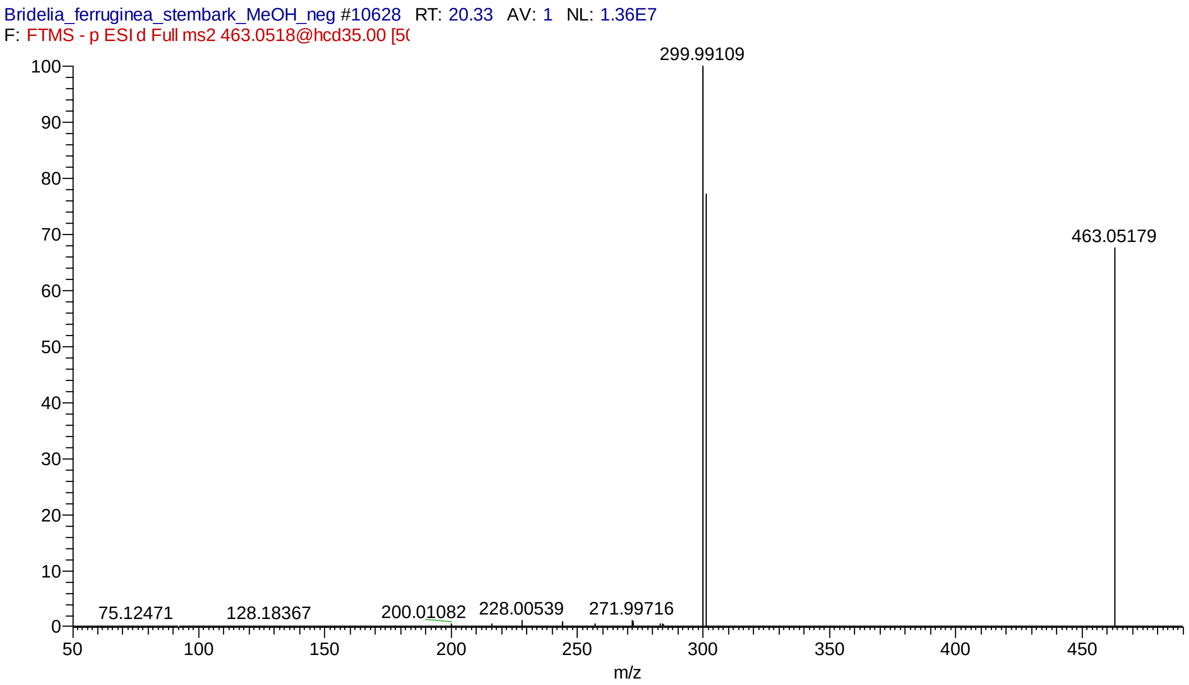

Figure S3. MS2 spectrum of Ellagic acid-O-hexoside m/z 463.05127 (ESI-)

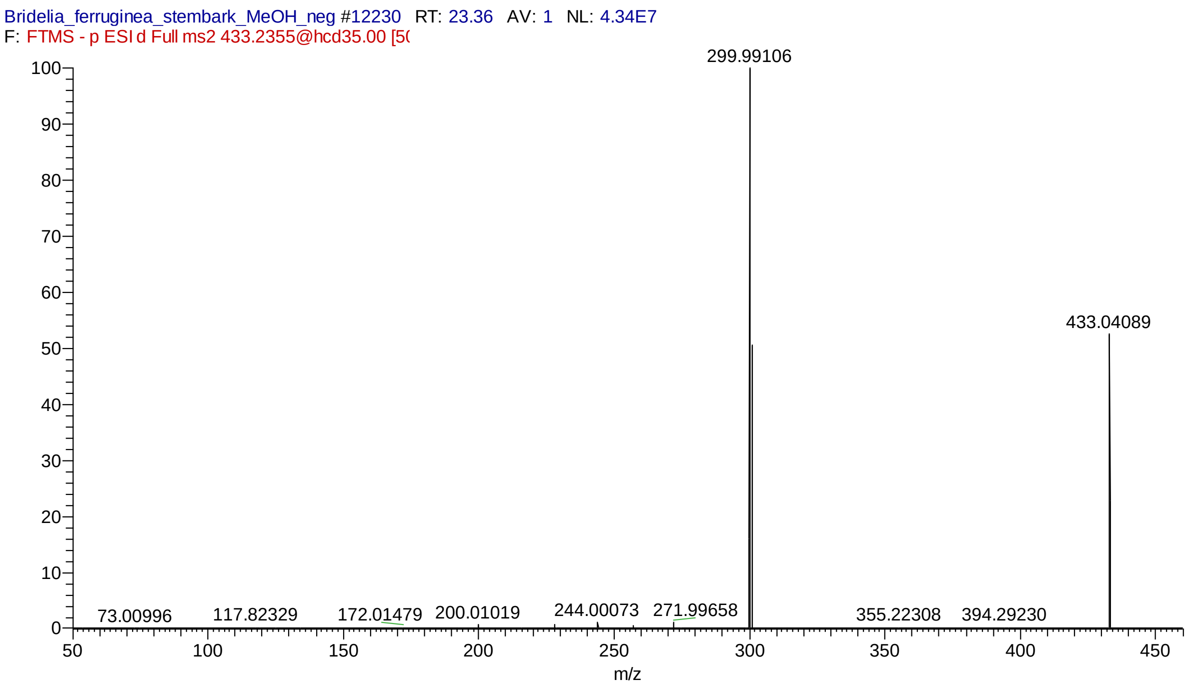

Figure S4. MS2 spectrum of Ellagic acid-O-pentoside m/z 433.04071 (ESI-)

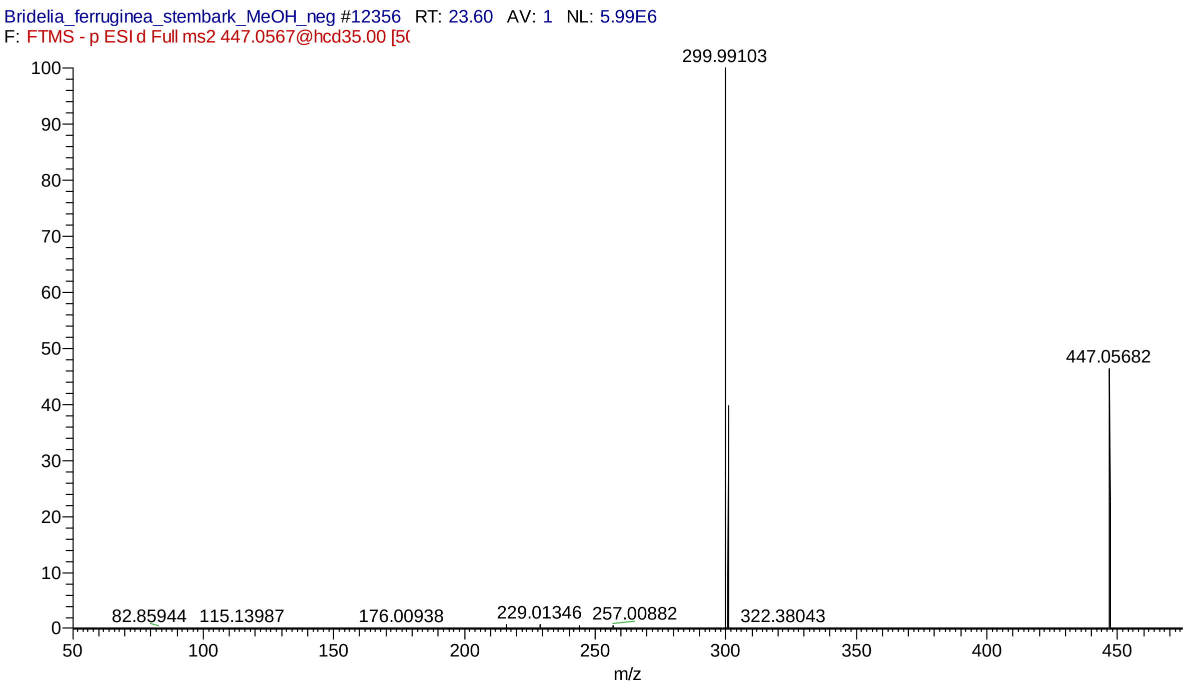

Figure S5. MS2 spectrum of Eschweilenol C (Ellagic acid-4-O-rhamnoside) m/z 447.05636 (ESI-)

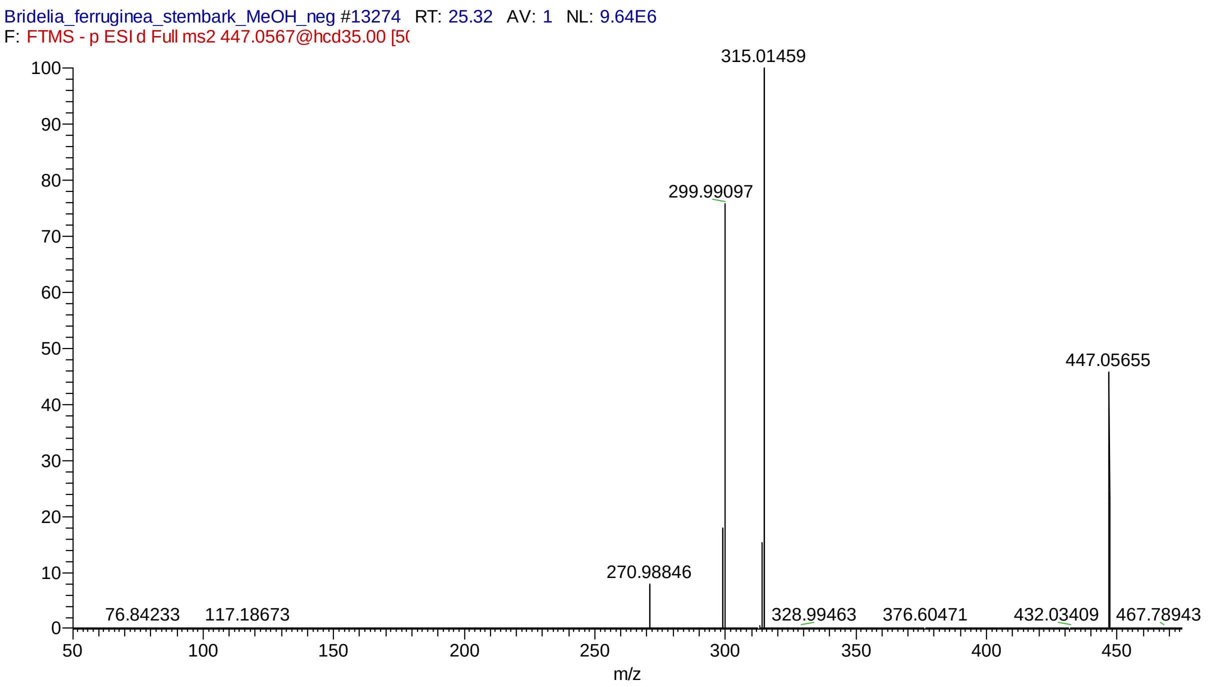

Figure S6. MS2 spectrum of Ducheside A (3-O-Methylellagic acid-4'-O-xyloside) m/z 447.05636 (ESI-)

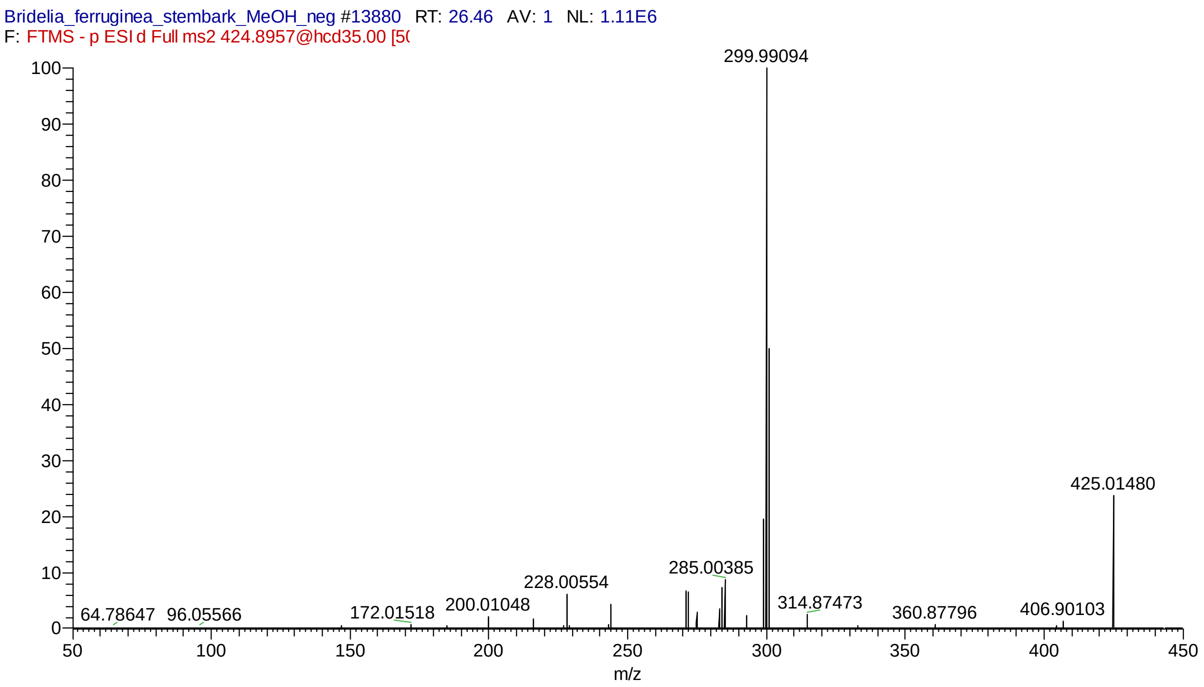

Figure S7. MS2 spectrum of Eschweilenol A or isomer m/z 425.01449 (ESI-)
